# Downregulation of miR-383 reduces depression-like behavior through targeting Wnt family member 2 (Wnt2) in rats

**DOI:** 10.1038/s41598-021-88560-6

**Published:** 2021-04-29

**Authors:** Shanshan Liu, Qing Liu, Yanjie Ju, Lei Liu

**Affiliations:** 1 Department of Psychiatry, Qingdao Mental Health Center, Qingdao University, No. 299 Nanjing Road, Qingdao City, 266000 Shandong Province People’s Republic of China; 2 Department of Psychology, Qingdao Mental Health Center, Qingdao University, Qingdao City, 266000 Shandong Province People’s Republic of China; 3 Department of Open Mental, Qingdao Mental Health Center, Qingdao University, Qingdao City, 266000 Shandong Province People’s Republic of China

**Keywords:** Psychology, Neuroscience, Molecular neuroscience

## Abstract

This study aimed to evaluate the role of miR-383 in the regulation of Wnt-2 signaling in the rat model of chronic stress. The male SD rats with depressive-like behaviors were stimulated with chronic unpredictable mild stress (CUMS) including ice-water swimming for 5 min, food deprivation for 24 h, water deprivation for 24 h, stimulating tail for 1 min, turning night into day, shaking for 15 min (once/s), and wrap restraint (5 min/time) every day for 21 days. The expression levels of miRNAs were detected by qRT-PCR, and the expression levels of Wnt2, depression-impacted proteins (GFAP, BDNF, CREB), brain neurotransmitters (5-HT, NE, DA) and apoptosis-related proteins (Bax and Bcl-2) were evaluated by qRT-PCR and western blot. Bioinformatic analysis and luciferase reporter assay were performed to determine the relationship between miR-383 and Wnt2. Ethological analysis was evaluated by sugar preference test, refuge island test and open field tests. Rescue experiments including knockdown of miR-383, overexpression and silencing of Wnt2 were performed to determine the role of miR-383. High expression levels of miR-383 were observed in the hippocampus of rats submitted to CUMS model. Downregulation of miR-383 significantly inhibited the apoptosis and inflammatory response of hippocampal neurons, and increased the expression levels of GFAP, BDNF and CREB which were impacted in depression, as well as neurotransmitters, then attenuated neural injury in rats induced by CUMS. Furthermore, Wnt family member 2 (Wnt2) was identified as a target of miR-383, and silencing of Wnt2 obviously attenuated the protective effect of miR-383 inhibitor on the apoptosis and inflammatory response in hippocampal neurons, as well as neural injury in CUMS-induced rats. Downregulation of miR-383 ameliorated the behavioral and neurochemical changes induced by chronic stress in rats by directly targeting Wnt2, indicating that the miR-383/Wnt2 axis might be a potential therapeutic target for MDD.

## Introduction

Major depressive disorder (MDD) is ranked by WHO as the single largest contributor to global disability, and hundreds of millions of people have suffered from this disease, which is characterized by health damage and even suicidal thoughts^1–3^. Current treatments of resistant depression remain largely empirical, and there are no bench-mark antidepressants^4^. In addition, a large number of patients with MDD fail to be response to traditional therapeutic strategy and always undergo relapse and obvious functional impairment^5^. Therefore, better understanding of molecular mechanisms will contribute to the development of new therapeutic targets for MDD in clinical application.

MicroRNAs (miRNAs) are a group of non-coding RNAs with 20 nucleotides in length, and function as crucial epigenetic regulators in human cells^6,7^. Extensive studies have revealed that a series of miRNAs are abnormally expressed in depression and may play important roles during the progression of MDD. For example, one study performed a microarray analysis and found that a total of 153 differentially expressed miRNAs were affected by chronic unpredictable mild stress (CUMS)^8^. MiR-124 was upregulated in prefrontal cortex of a CUMS model, and overexpression of miR-124 exacerbated depression-like behaviors^9^. These reports highlighted the essential roles of miRNAs in depression. One recent study revealed that miR-383 and its potential binding genes played crucial roles during the development of rumination based on a genome-wide association study^10^. Another study found that miR-383 was significantly upregulated in cortical glutamatergic neurons during spinal injury response^11^. These evidences suggested that miR-383 may play essential roles in the progression of psychiatric diseases. However, the function of miR-383 in MDD remains unclear.

Wnt family proteins, a group of secretory glycoproteins, have been reported to play essential roles in the development of nervous system^12^. Wnt2 belongs to the Wnt family and shows potent anti-depressant effects and can inhibit the development of MDD^13^. Wnt2 can activate CREB, and phosphorylated CREB (p-CREB) can promote the expression of BDNF, which is a downstream gene of CREB^14,15^. Previous studies reported that the activation of p-CREB/BDNF pathway could efficiently protect against neuronal apoptosis^16,17^. Glial fibrillary acidic protein (GFAP) is an intermediate filament protein that is primarily expressed in astrocytes, and was markedly upregulated in MDD^18^. These key genes are closely involved in the progression of MDD, and miR-221 has been demonstrated to control the progression of MDD in chronic unpredictable mild stress (CUMS) rats by regulating the Wnt2/CREB/BDNF signaling in hippocampal neurons^19^.

Here, we demonstrated that miR-383 was markedly upregulated in the hippocampal tissues of rats submitted to CUMS. Downregulation of miR-383 inhibited the apoptosis of hippocampal neurons and inflammatory response, meanwhile increased the expression levels of neurotransmitters including 5-HT, NE and DA. Furthermore, Wnt2 was identified as a target of miR-383, and silencing of Wnt2 could dramatically reverse the protective effect of miR-383 inhibitor in a CUMS rat model. Taken together, our results provided a novel regulatory axis of Wnt2, specifically, miR-383 might promote the progression of MDD through targeting and inhibiting the expression of Wnt2.

## Results

### Wnt2 was downregulated and miR-383 was upregulated in hippocampi tissues of rats submitted to CUMS

**Figure 1:**
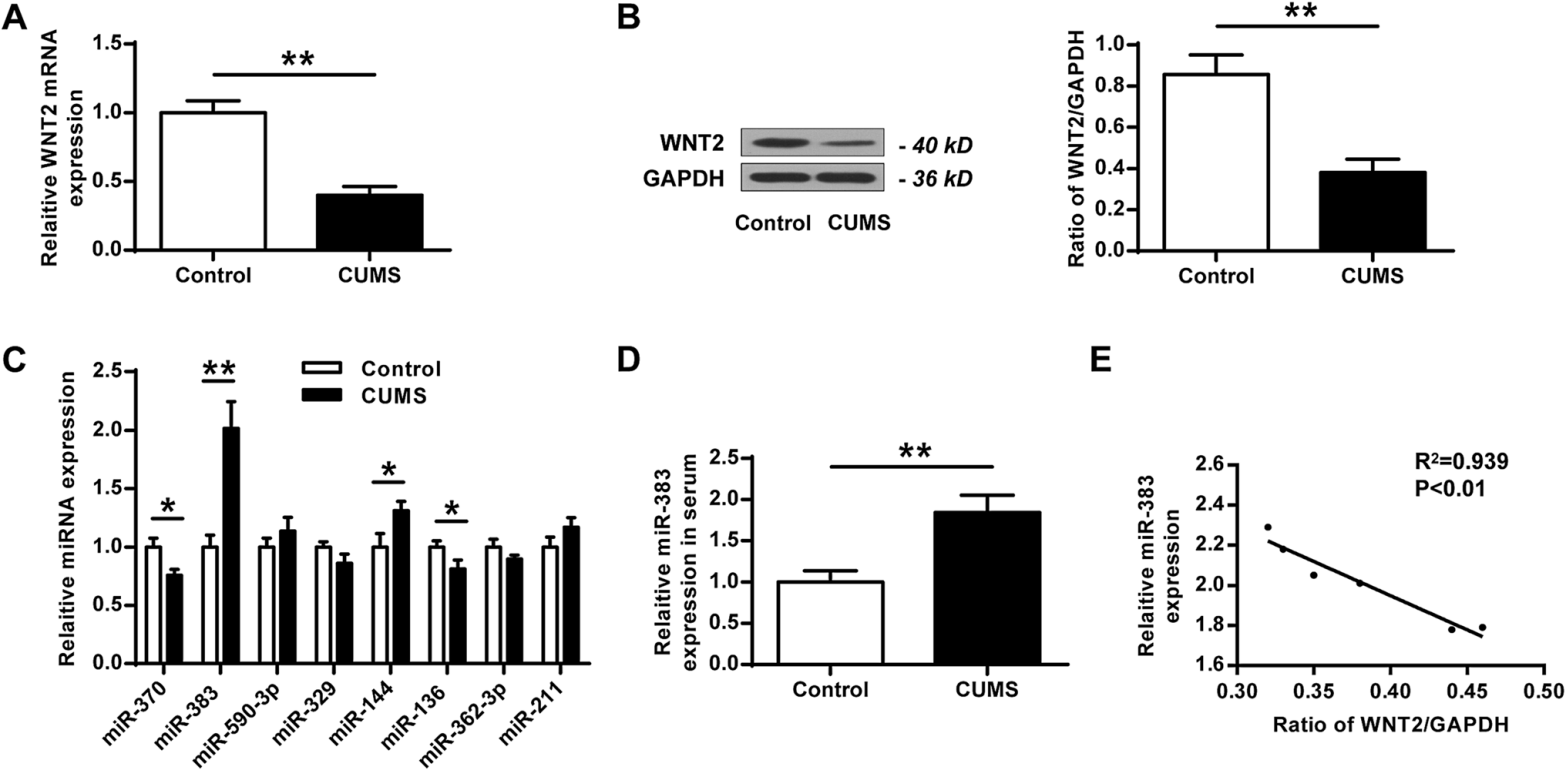
WNT2 was downregulated and miR-383 was upregulated in hippocampi tissues of chronic unpredictable mild stress (CUMS)-induced rats. (**A**,**B**) The mRNA level (**A**) and protein level (**B**) of Wnt2 in hippocampal tissues of CUMS-induced rats and control group. (**C**) The mRNA level of miRNAs in hippocampal tissues of CUMS-induced rats and control group. (**D**) The mRNA level of miR-383 in hippocampal tissues of CUMS-induced rats and control group. (**E**) The correlation between miR-383 level and Wnt2 level was evaluated by Pearson’s analysis. N = 6. Data were presented as mean ± SD and each experiment was repeated three times. *P < 0.05, **P < 0.01.

It has been reported that Wnt2 has antidepressant effects in the CUMS model^13^. To explore the regulatory network of Wnt2, CUMS was used to induce MDD rat model. It showed that Wnt2 was significantly downregulated in hippocampi tissues of rats submitted to CUMS compared with that in the control group at both mRNA (t test, t_(10)_ = 9.820, *P* = 0.001) (Fig. 1A) and protein (t test, t_(10)_ = 7.150, *P* = 0.002) levels (Fig. 1B). Bioinformatic analysis revealed that there were several miRNAs that might be potential regulators of Wnt2, including miR-370, miR-383, miR-590-3p, miR-329, miR-144, miR-139, miR-362-3p and miR-211. qRT-PCR results showed that only miR-383 was significantly upregulated in hippocampi tissues of rats submitted to CUMS compared with that in the control group (t test, t_(10)_ = − 7.003, *P* = 0.002) (Fig. 1C). Meanwhile, miR-383 was also significantly upregulated in serum of rats submitted to CUMS in comparison to that in the control group (t test, t_(10)_ = − 5.810, *P* = 0.004) (Fig. 1D). Moreover, Pearson’s analysis revealed that the expression of miR-383 was negatively correlated to the expression of Wnt2 in hippocampi tissues of rats submitted to CUMS (R^2^ = 0.939, *P* = 0.001) (Fig. 1E). These results indicated that miR-383 might play important roles in MDD.

### MiR-383 served as a ceRNA of Wnt2

**Figure 2:**
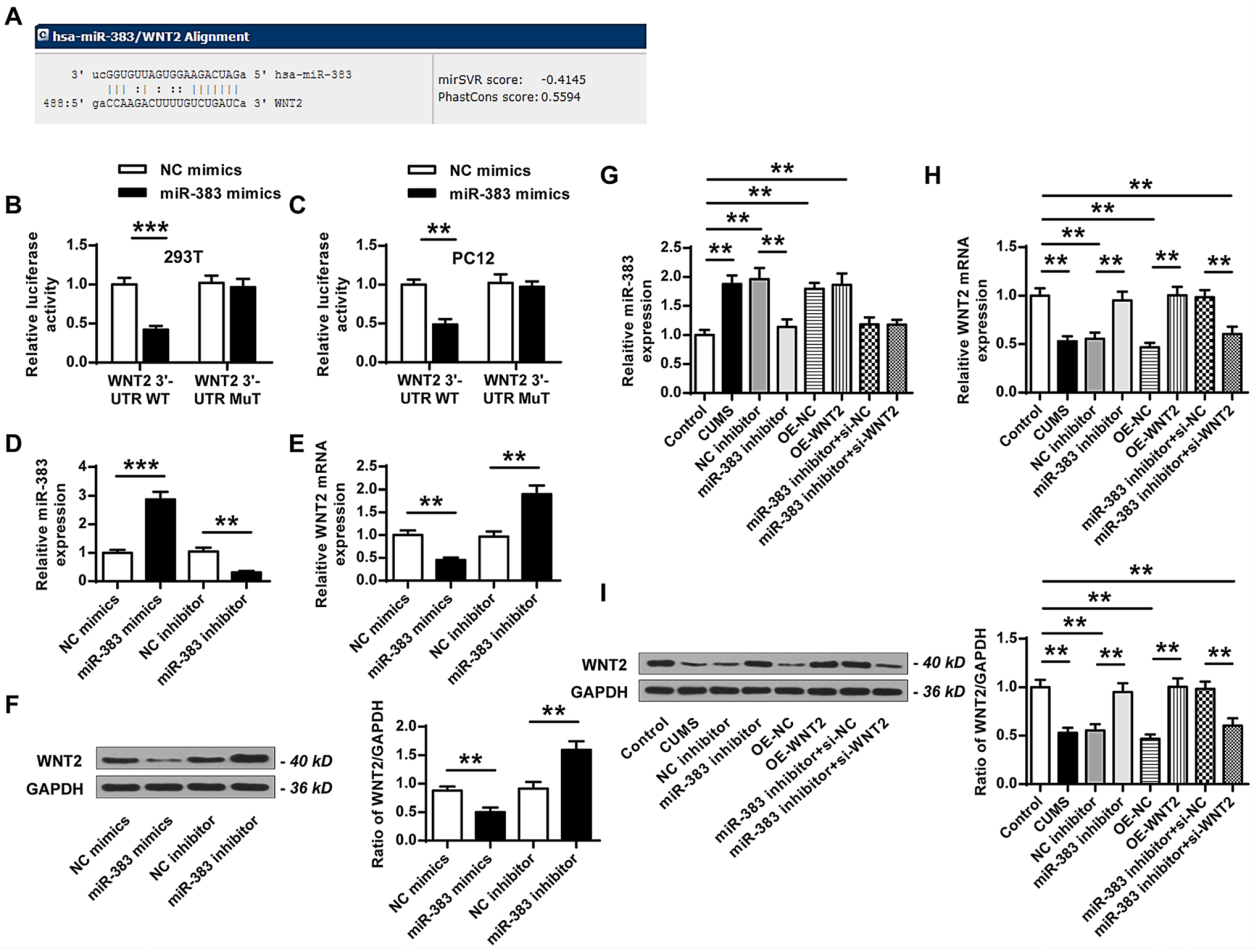
WNT2 was a target of miR-383. (**A**) The results of biological prediction between miR-383 and Wnt2. (**B**,**C**) The relative luciferase activity of WT or MUT 3′-UTR of Wnt2 in 293 T cells (**B**) and PC12 cells (**C**) transfected with miR-383 mimics or NC mimics was evaluated by the dual luciferase reporter system (n = 3). (**D**–**F**) The PC12 cells were transfected with NC mimics, miR-383 mimics, NC inhibitor or miR-383 inhibitor. (**D**) Transfection efficiency of miR-383 mimics/inhibitor in the PC12 cells was evaluate by qRT-PCR (n = 3). (**E**) The mRNA level of Wnt2 in PC12 cells was detected by qRT-PCR (n = 3). (**F**) The protein level of Wnt2 in PC12 cells was evaluated by western blot (n = 3). (**G**–**I**) Rats were injected with miR-383 inhibitor, NC inhibitor, OE-NC (the negative control of Wnt2 overexpression), OE-Wnt2 (Wnt2 overexpression), miR-383 inhibitor plus si-NC (the negative control of Wnt2 silencing) or miR-383 inhibitor plus si-Wnt2 (Wnt2 silencing), and then submitted to CUMS. (**G**) The mRNA level of miR-383 in hippocampal tissues was evaluated by qRT-PCR (n = 6). (**H**,**I**) The mRNA level (**H**) and protein level (**I**) in hippocampal tissues were evaluated by western blot (n = 6). Data were presented as mean ± SD and each experiment was repeated three times. **P < 0.01, ***P < 0.001.

To explore the specific mechanism between miR-383 and Wnt2, the prediction website (http://www.microrna.org/microrna/home.do) was used to predict their interaction region. The results showed that miR-383 had a potential binding site in the 3′-UTR of Wnt2 (Fig. 2A). To validate this interaction, the luciferase reporter plasmids containing WT or MUT 3′-UTR of Wnt2 were co-transfected with miR-383 mimics (overexpression of miR-383) or NC mimics (miR-383 mimics negative control) into 293 T and PC12 cells. The luciferase reporter assay results indicated that miR-383 mimics significantly decreased the relative luciferase activity of WT 3′-UTR of Wnt2 (Fig. 2B, ANOVA, F_(3,8)_ = 34.586, *P* = 0.000; LSD test, t = 10.339, *P* = 0.000; Fig. 2C, ANOVA, F_(3,8)_ = 30.466, *P* = 0.000; LSD test, t = 9.253, *P* = 0.001), and exhibited no obvious change on MUT 3′-UTR of Wnt2 compared with NC mimics in both 293Ts cell (Fig. 2B) and PC12 cells (Fig. 2C). Meanwhile, qRT-PCR results showed that miR-383 mimics significantly increased the expression levels of miR-383 compared with NC mimics (ANOVA, F_(3,8)_ = 142.393, *P* = 0.000; LSD test, t = − 11.440, *P* = 0.000), and miR-383 inhibitor (knockdown of miR-383) decreased the expression levels of miR-383 compared with NC inhibitor (negative control) (ANOVA, F_(3,8)_ = 142.393, *P* = 0.000; LSD test, t = 8.814, *P* = 0.001) (Fig. 2D). Moreover, the expression levels of Wnt2 were significantly decreased in miR-383 mimics group compared with that in NC mimics at both mRNA and protein levels (Fig. 2E, ANOVA, F_(3,8)_ = 74.391, *P* = 0.000; LSD test, t = 8.326, *P* = 0.001; Fig. 2F, ANOVA, F_(3,8)_ = 50.501, *P* = 0.000; LSD test, t = 6.034, *P* = 0.004), while increased in miR-383 inhibitor group compared with that in NC inhibitor group (Fig. 2E, ANOVA, F_(3,8)_ = 74.391, *P* = 0.000; LSD test, t = − 7.592, *P* = 0.002; Fig. 2F, ANOVA, F_(3,8)_ = 50.501, *P* = 0.000; LSD test, t = − 6.101, *P* = 0.004). Furthermore, the rats were injected with miR-383 inhibitor, NC inhibitor, OE-NC (the negative control of Wnt2 overexpression), OE-Wnt2 (Wnt2 overexpression), miR-383 inhibitor plus si-NC (the negative control of Wnt2 silencing) or miR-383 inhibitor plus si-Wnt2 (Wnt2 silencing), and then submitted to CUMS. Then the expression levels of miR-383 were evaluated after rats were recovered for 1 week and the results indicated that miR-383 inhibitor could significantly decrease the expression levels of miR-383 in comparison to that in NC inhibitor group (ANOVA, F_(7,40)_ = 25.858, *P* = 0.000; LSD test, t = 6.163, *P* = 0.004), while no obvious change was observed in other groups (Fig. 2G). In addition, the expression levels of Wnt2 in these groups were also measured and the results showed that miR-383 inhibitor significantly increased the expression levels of Wnt2 compare with that in NC inhibitor group (Fig. 2H, ANOVA, F_(7,40)_ = 33.815, *P* = 0.000; LSD test, t = − 6.187, *P* = 0.003; Fig. 2I, ANOVA, F_(7,40)_ = 49.170, *P* = 0.000; LSD test, t = − 8.561, *P* = 0.001), OE-Wnt2 increased the expression levels of Wnt2 compared with that in OE-NC group (Fig. 2H, ANOVA, F_(7,40)_ = 33.815, *P* = 0.000; LSD test, t = − 9.554, *P* = 0.001; Fig. 2I, ANOVA, F_(7,40)_ = 49.170, *P* = 0.000; LSD test, t = − 9.684, *P* = 0.001), and miR-383 inhibitor plus si-Wnt2 significantly reversed miR-383 inhibitor enhanced expression of Wnt2 at both mRNA and protein levels (Fig. 2H, ANOVA, F_(7,40)_ = 33.815, *P* = 0.000; LSD test, t = 6.146, *P* = 0.004; Fig. 2I, ANOVA, F_(7,40)_ = 49.170, *P* = 0.000; LSD test, t = 7.976, *P* = 0.001). These results indicated that Wnt2 was a target of miR-383.

### Downregulation of miR-383 or upregulation of Wnt2 could reduce CUMS-induced depression-like behaviors

**Figure 3:**
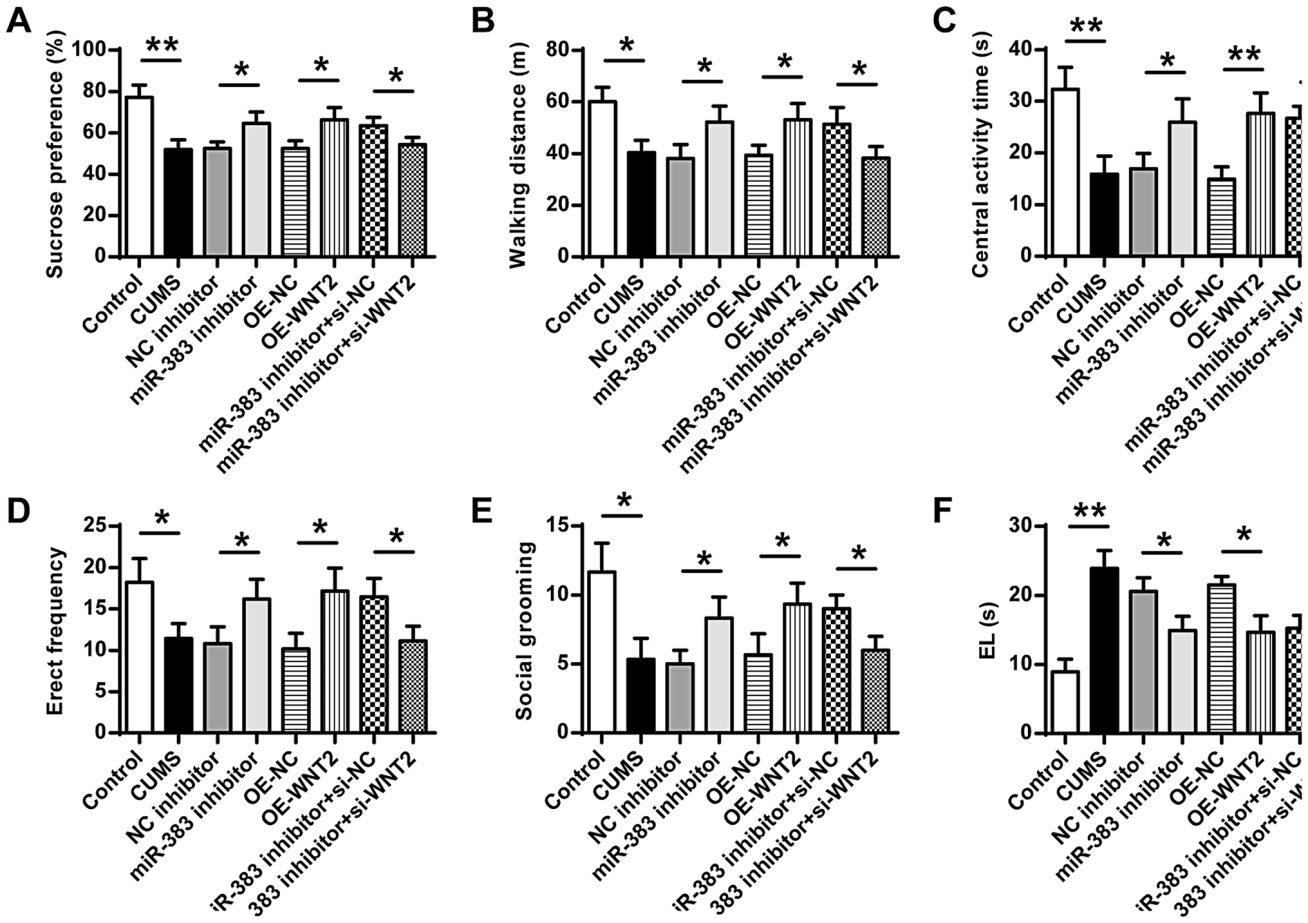
Downregulation of miR-383 or upregulation of Wnt2 could reduce CUMS-induced depression-like behaviors in rats. The sucrose preference (**A**), walking distance (**B**), central activity time (**C**), Erect frequency (**D**), Social grooming (**E**) and Escape latency (EL) (**F**) of rats in different groups. N = 6. Data were presented as mean ± SD and each experiment was repeated three times. *P < 0.05, **P < 0.01.

The specific roles of miR-383 and Wnt2 in the development of depression-like behaviors of CUMS-induced rats were then explored. The sucrose preference test results showed that the sucrose consumption rate was decreased in CUMS-induced rats compared with that in the control group (ANOVA, F_(7,40)_ = 11.073, *P* = 0.000; LSD test, t = 5.759, *P* = 0.005), increased in the miR-383 inhibitor group compared with that in NC inhibitor group (ANOVA, F_(7,40)_ = 11.073, *P* = 0.000; LSD test, t = − 3.284, *P* = 0.030), increased in the OE-Wnt2 group compared with that in OE-NC group (ANOVA, F_(7,40)_ = 11.073, *P* = 0.000; LSD test, t = − 3.350, *P* = 0.029), and also decreased in miR-383 inhibitor plus si-Wnt2 group compared with that in miR-383 inhibitor plus si-NC group (ANOVA, F_(7,40)_ = 11.073, *P* = 0.000; LSD test, t = 2.933, *P* = 0.043) (Fig. 3A). Based on the tests of walking distance (Fig. 3B), central activity time (Fig. 3C), Erect frequency (Fig. 3D), social grooming (Fig. 3E) and escape latency (EL) (Fig. 3F), except for the increase of EL, other indexes were all reduced in CUMS-induced rats compared with that in the control group (Fig. 3B, ANOVA, F_(7,40)_ = 7.309, *P* = 0.000; LSD test, t = 4.602, *P* = 0.010; Fig. 3C, ANOVA, F_(7,40)_ = 11.132, *P* = 0.000; LSD test, t = 5.188, *P* = 0.007; Fig. 3D, ANOVA, F_(7,40)_ = 6.478, *P* = 0.001; LSD test, t = 3.456, *P* = 0.026; Fig. 3E, ANOVA, F_(7,40)_ = 8.271, *P* = 0.001; LSD test, t = 4.249, *P* = 0.013; Fig. 3F, ANOVA, F_(7,40)_ = 16.725, *P* = 0.001; LSD test, t = − 8.139, *P* = 0.001). And except for the decrease of EL, other indexes were all increased in miR-383 inhibitor group compared with that in NC inhibitor group (Fig. 3B, ANOVA, F_(7,40)_ = 7.309, *P* = 0.000; LSD test, t = − 2.941, *P* = 0.042; Fig. 3C, ANO VA, F_(7,40)_ = 11.132, *P* = 0.000; LSD test, t = − 2.901, *P* = 0.044; Fig. 3D, ANOVA, F_(7,40)_ = 6.478, *P* = 0.001; LSD test, t = − 2.933, *P* = 0.043; Fig. 3E, ANOVA, F_(7,40)_ = 8.271, *P* = 0.001; LSD test, t = − 3.162, *P* = 0.034; Fig. 3F, ANOVA, F_(7,40)_ = 16.725, *P* = 0.001; LSD test, t = 3.441, *P* = 0.026). Expect for the decrease of EL, other indexes were all increased in OE-Wnt2 group compared with that in OE-NC group (Fig. 3B, ANOVA, F_(7,40)_ = 7.309, *P* = 0.000; LSD test, t = − 3.247, *P* = 0.031; Fig. 3C, ANOVA, F_(7,40)_ = 11.132, *P* = 0.000; LSD test, t = − 4.737, *P* = 0.009; Fig. 3D, ANOVA, F_(7,40)_ = 6.478, *P* = 0.001; LSD test, t = − 3.565, *P* = 0.023; Fig. 3E, ANOVA, F_(7,40)_ = 8.271, *P* = 0.001; LSD test, t = − 2.940, *P* = 0.042; Fig. 3F, ANOVA, F_(7,40)_ = 16.725, *P* = 0.001; LSD test, t = 4.385, *P* = 0.012). Meanwhile, expect for the increase of EL, other indexes were all decreased in miR-383 inhibitor plus si-WNT2 group compared with that in miR-383 inhibitor plus si-NC group (Fig. 3B, ANOVA, F_(7,40)_ = 7.309, *P* = 0.000; LSD test, t = 2.873, *P* = 0.045; Fig. 3C, ANOVA, F_(7,40)_ = 11.132, *P* = 0.000; LSD test, t = 4.171, *P* = 0.014; Fig. 3D, ANOVA, F_(7,40)_ = 6.478, *P* = 0.001; LSD test, t = 3.213, *P* = 0.032; Fig. 3E, ANOVA, F_(7,40)_ = 8.271, *P* = 0.001; LSD test, t = 3.674, *P* = 0.021; Fig. 3F, ANOVA, F_(7,40)_ = 16.725, *P* = 0.001; LSD test, t = − 2.974, *P* = 0.041). In addition, to explore whether knockdown of miR-383 affected the depression-like behaviors in normal rats, rats were injected with miR-383 inhibitor or NC inhibitor with no CUMS treatment. The ethological examination indicated that: compared with the control group and inhibitor NC group, there was no obvious change in sucrose preference, walking distance, central activity time, erect frequency, social grooming in the miR-383 inhibitor group. However, rats in miR-382 inhibitor group showed a decreased El compared with that in the control group and inhibitor NC group (Supplementary Fig. 1). All these results indicated that downregulation of miR-383 efficiently reduced CUMS-induced depression-like behaviors in rats by targeting Wnt2.

### Downregulation of miR-383 or upregulation of Wnt2 attenuated cell injury and apoptosis of hippocampal neurons

**Figure 4:**
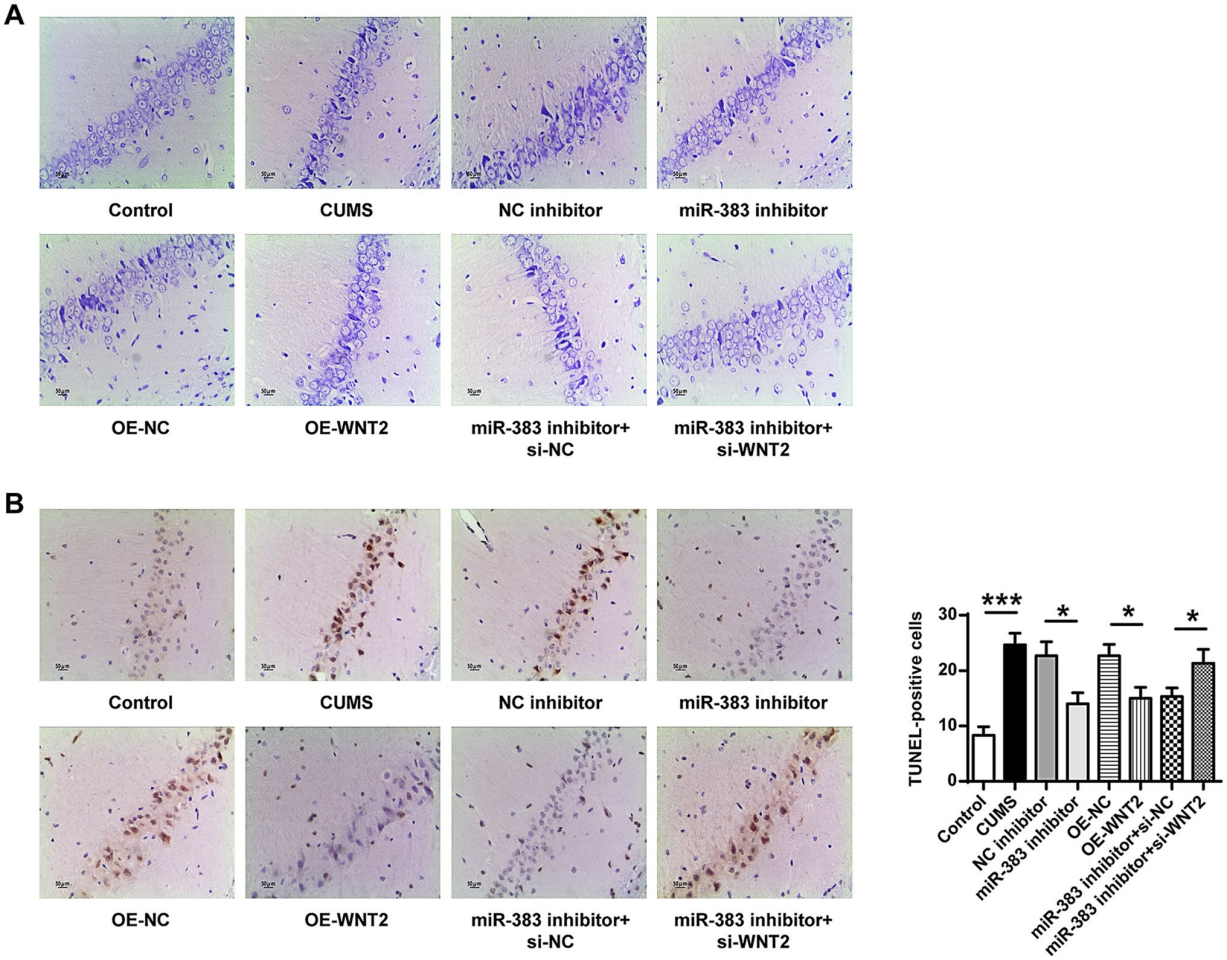
Downregulation of miR-383 or upregulation of Wnt2 attenuated cell injury and apoptosis rate of hippocampal neurons in CUMS-induced rats. (**A**) Images of Nissl’s staining in rat hippocampi tissues of different groups. Magnification, × 200; scale bar = 50 μm. (**B**) The apoptosis of rat hippocampal neurons of different groups was detected by TUNEL staining. Magnification, × 200; scale bar = 50 μm. N = 6. Data were presented as mean ± SD and each experiment was repeated three times. *P < 0.05, ***P < 0.001.

The Nissl’s staining was used to evaluate the morphological changes of hippocampal neurons in different groups and the results were shown in Fig. 4A. Compared with the control group, hippocampal pyramidal neurons of rats in CUMS-induced rats exhibited larger intercellular space, decreased volume and processes as well as attenuated cell layer. The injury of hippocampal pyramidal neurons in miR-383 inhibitor group, OE-Wnt2 group and miR-383 inhibitor plus si-NC group was ameliorated compared with their corresponding control groups. Meanwhile, TUNEL staining was performed to evaluate the apoptosis rate of hippocampal neurons in different groups and the results were shown in Fig. 4B. The numbers of TUNEL positive cells in CUMS-induced rats were obviously increased compared with that in the control group (ANOVA, F_(7,40)_ = 22.655, *P* = 0.000; LSD test, t = − 10.957, *P* = 0.000); The TUNEL positive cells in miR-383 inhibitor group were significantly decreased compared with that in NC inhibitor group (ANOVA, F_(7,40)_ = 22.655, *P* = 0.000; LSD test, t = 4.670, *P* = 0.010); Compared to OE-NC group, the TUNEL positive cells were reduced in the OE-Wnt2 group (ANOVA, F_(7,40)_ = 22.655, *P* = 0.000; LSD test, t = 4.600, *P* = 0.010). In addition, the TUNEL positive cells were significantly increased in miR-383 inhibitor plus si-Wnt2 group compared with that in miR-383 inhibitor plus si-NC group (ANOVA, F_(7,40)_ = 22.655, *P* = 0.000; LSD test, t = − 3.530, *P* = 0.024). These results demonstrated that downregulation of miR-383 or upregulation of Wnt2 could efficiently attenuate cell injury and apoptosis of hippocampal neurons in CUMS-induced rats.

### Downregulation of miR-383 or upregulation of Wnt2 increased the expression levels of GFAP in hippocampal tissues of CUMS-induced rats

**Figure 5:**
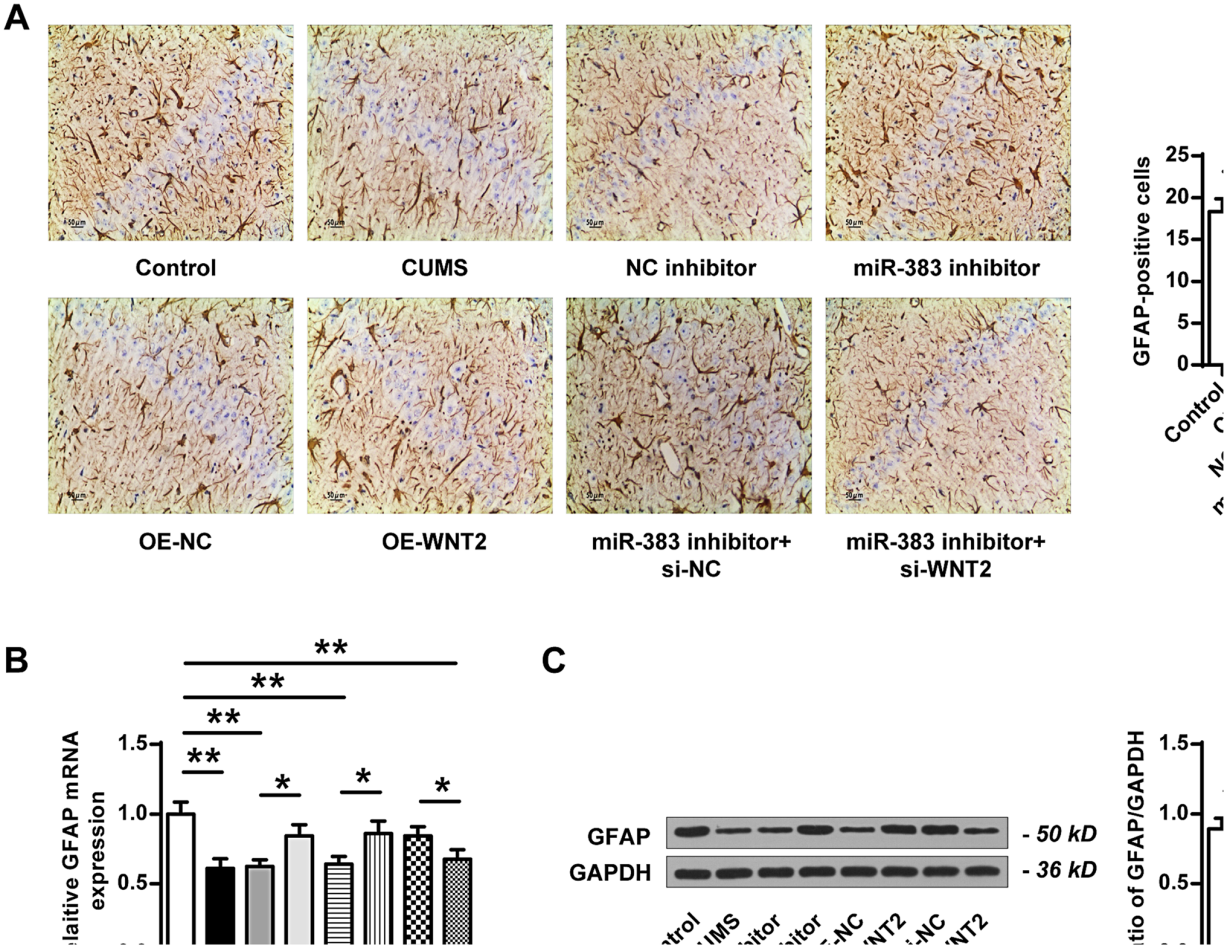
Downregulation of miR-383 or upregulation of Wnt2 increased the expression levels of GFAP in the hippocampal tissues of CUMS-induced rats. (**A**) Images of immunohistochemical staining for GFAP-positive cells in hippocampal tissues. Magnification, × 200; scale bar = 50 μm. (**B**,**C**) The mRNA level (**B**) and protein level (**C**) of GFAP in hippocampal tissues of rats in different groups. N = 6. Data were presented as mean ± SD and each experiment was repeated three times. *P < 0.05, **P < 0.01.

Immunohistochemical staining was performed to evaluate the expression of GFAP in hippocampal tissues of different groups and the results were shown in Fig. 5A. In CUMS group, the GFAP-positive cells were significantly decreased compared with that in the control group (ANOVA, F_(7,40)_ = 10.786, *P* = 0.000; LSD test, t = 5.060, *P* = 0.007). Compared to NC inhibitor group, the positive expression of GFAP was obviously elevated in the miR-383 inhibitor group (ANOVA, F_(7,40)_ = 10.786, *P* = 0.000; LSD test, t = − 4.111, *P* = 0.015). Similarly, the GFAP-positive cells were significantly increased in OE-Wnt2 group compared with that in OE-NC group (ANOVA, F_(7,40)_ = 10.786, *P* = 0.000; LSD test, t = − 3.674, *P* = 0.021). In addition, the GFAP-positive cells in miR-383 inhibitor plus si-Wnt2 group were significantly decreased compared with that in miR-383 inhibitor plus si-NC group (ANOVA, F_(7,40)_ = 10.786, *P* = 0.000; LSD test, t = 3.182, *P* = 0.033). Meanwhile, the level of GFAP in different groups was detected by qRT-PCR (Fig. 5B) and western blot (Fig. 5C). It showed that the expression of GFAP was significantly downregulated in CUMS group compared with that in the control group (Fig. 5B, ANOVA, F_(7,40)_ = 12.089, *P* = 0.000; LSD test, t = 6.116, *P* = 0.004; Fig. 5C, ANOVA, F_(7,40)_ = 14.001, *P* = 0.000; LSD test, t = 7.107, *P* = 0.002); The expression levels of GFAP were obviously increased in miR-383 inhibitor group compared with that in NC inhibitor group (Fig. 5B, ANOVA, F_(7,40)_ = 12.089, *P* = 0.000; LSD test, t = − 4.070, *P* = 0.015; Fig. 5C, ANOVA, F_(7,40)_ = 14.001, *P* = 0.000; LSD test, t = − 4.228, *P* = 0.013). The expression levels of GFAP were also increased in OE-Wnt2 group compared with that in OE-NC group (Fig. 5B, ANOVA, F_(7,40)_ = 12.089, *P* = 0.000; LSD test, t = − 3.601, *P* = 0.023; Fig. 5C, ANOVA, F_(7,40)_ = 14.001, *P* = 0.000; LSD test, t = − 3.543, *P* = 0.024). Compared to the miR-383 inhibitor plus si-NC group, the expression levels of GFAP were declined in the miR-383 inhibitor plus si-Wnt2 group (Fig. 5B, ANOVA, F_(7,40)_ = 12.089, *P* = 0.000; LSD test, t = 3.101, *P* = 0.036; Fig. 5C, ANOVA, F_(7,40)_ = 14.001, *P* = 0.000; LSD test, t = 4.447, *P* = 0.011). These results demonstrated that downregulation of miR-383 or upregulation of Wnt2 could significantly increase the expression levels of GFAP in hippocampal tissues of CUMS-induced rats.

### Downregulation of miR-383 or upregulation of Wnt2 increased the expression levels of CREB, BDNF and Bcl-2, and decreased the expression levels of Bax

**Figure 6:**
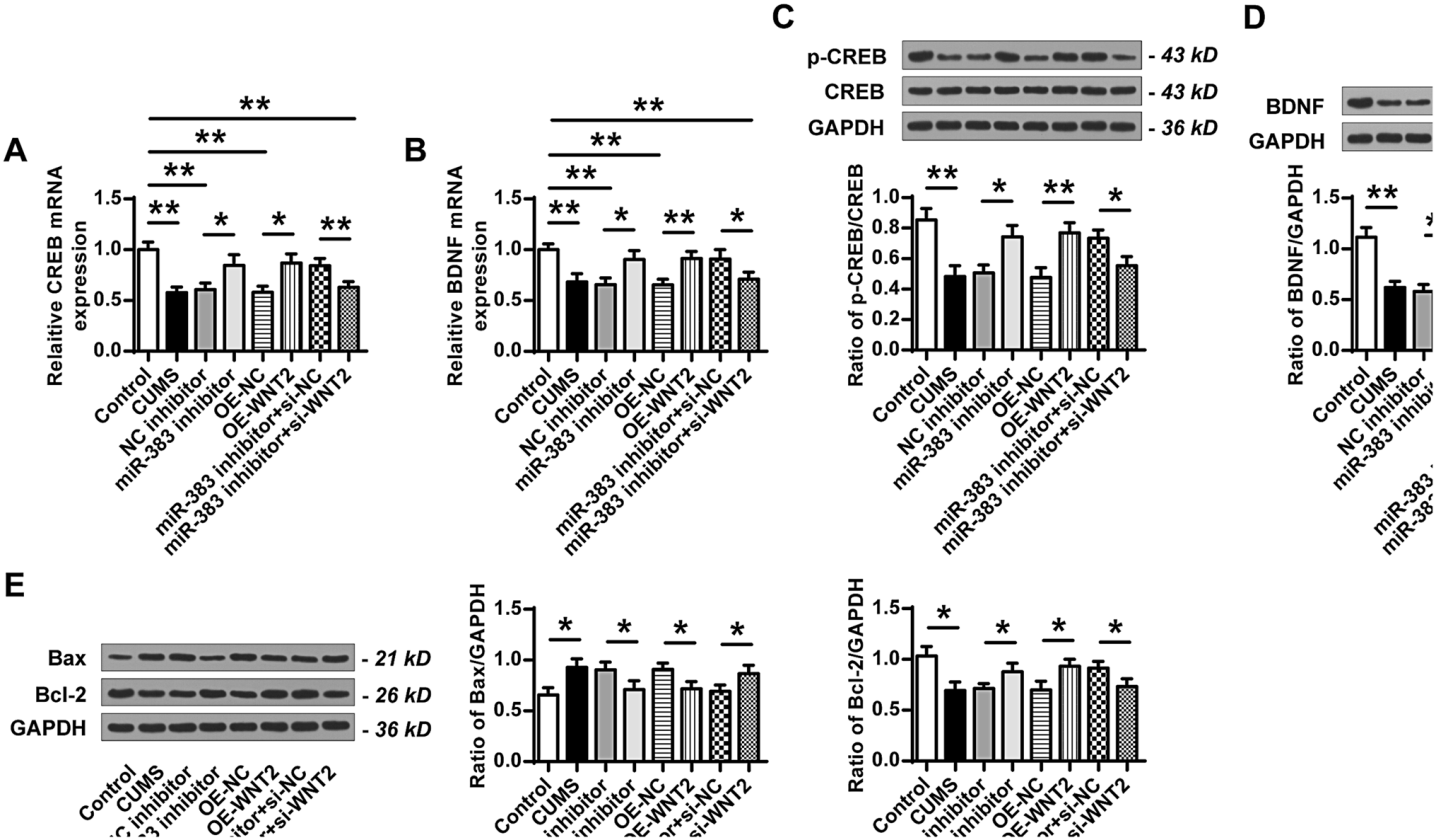
Downregulation of miR-383 or upregulation of Wnt2 increased the expression levels of CREB, BDNF and Bcl-2, while decreased the expression levels of Bax in the hippocampal tissues of CUMS-induced rats. (**A**,**B**) The mRNA levels of CREB (**A**) and BDNF (**B**) in hippocampal tissues of rats in different groups were evaluated by qRT-PCR. (**C**,**D**) The protein levels of p-CREB (**C**) and BDNF (**D**) in hippocampal tissues of rats in different groups were evaluated by western blot. (**E**) The protein levels of Bcl-2 and Bax in rat hippocampal tissues of rats in different groups were evaluated by western blot. N = 6. Data were presented as mean ± SD and each experiment was repeated three times. *P < 0.05, **P < 0.01.

Moreover, the mRNA level of CREB (Fig. 6A) and the protein level of p-CREB (Fig. 6C) in the hippocampal tissues of rats were evaluated. The results showed that the mRNA level of CREB and the protein level of p-CREB were decreased in other groups compared with that in the control group (mRNA level: CUMS vs Control, ANOVA, F_(7,40)_ = 14.784, *P* = 0.000; LSD test, t = 7.846, *P* = 0.001; NC inhibitor vs Control, ANOVA, F_(7,40)_ = 14.784, *P* = 0.000; LSD test, t = 6.836, *P* = 0.002; OE-NC vs Control, ANOVA, F_(7,40)_ = 14.784, *P* = 0.000; LSD test, t = 7.543, *P* = 0.002; miR-383 inhibitor + si-WNT2 vs Control, ANOVA, F_(7,40)_ = 14.784, *P* = 0.000; LSD test, t = 6.832, *P* = 0.002. Protein level: CUMS vs Control, ANOVA, F_(7,40)_ = 15.949, *P* = 0.000; LSD test, t = 6.234, *P* = 0.003; NC inhibitor vs Control, ANOVA, F_(7,40)_ = 15.949, *P* = 0.000; LSD test, t = 6.604, *P* = 0.003; OE-NC vs Control, ANOVA, F_(7,40)_ = 15.949, *P* = 0.000; LSD test, t = 6.568, *P* = 0.003; miR-383 inhibitor + si-WNT2 vs Control, ANOVA, F_(7,40)_ = 15.949, *P* = 0.000; LSD test, t = 5.398, *P* = 0.006). Compared to the control group, the expression levels of CREB were reduced in the CUMS group (ANOVA, F_(7,40)_ = 15.949, *P* = 0.000; LSD test, t = 6.234, *P* = 0.003). Compared with the NC inhibitor group, the expression levels of CREB were increased in the miR-383 inhibitor group (ANOVA, F_(7,40)_ = 15.949, *P* = 0.000; LSD test, t = − 4.509, *P* = 0.011). Compared to the OE-NC group, the expression levels of CREB were enhanced in the OE-Wnt2 group (ANOVA, F_(7,40)_ = 15.949, *P* = 0.000; LSD test, t = − 5.500, *P* = 0.005). Compared to the miR-383 inhibitor plus si-NC group, the expression levels of CREB were lowered in the miR-383 inhibitor plus si-Wnt2 group (ANOVA, F_(7,40)_ = 15.949, *P* = 0.000; LSD test, t = 3.818, *P* = 0.019). The expression of BDNF was similar to CREB in hippocampi tissues of rats in different groups (Fig. 6B,D). In addition, the protein expression levels of Bcl-2 and Bax in hippocampi tissues of different groups were also detected by western blot (Fig. 6E). It showed that the protein expression levels of Bax were increased and Bcl-2 was decreased in CUMS group compared with that in the control group (Bax: ANOVA, F(7,40) = 7.013, *P* = 0.001; LSD test, t = − 4.222, *P* = 0.013). Bcl-2: ANOVA, F_(7,40)_ = 8.544, *P* = 0.000; LSD test, t = 4.617, *P* = 0.010). And the protein expression levels of Bax were decreased and Bcl-2 were increased in miR-383 inhibitor group compared with that in NC inhibitor group (Bax: ANOVA, F_(7,40)_ = 7.013, *P* = 0.001; LSD test, t = 2.922, *P* = 0.043. Bcl-2: ANOVA, F_(7,40)_ = 8.544, *P* = 0.000; LSD test, t = − 2.908, *P* = 0.044). The protein expression levels of Bax were decreased and Bcl-2 were increased in OE-Wnt2 group compared with that in OE-NC group (Bax: ANOVA, F_(7,40)_ = 7.013, *P* = 0.001; LSD test, t = 3.625, *P* = 0.022. Bcl-2: ANOVA, F_(7,40)_ = 8.544, *P* = 0.000; LSD test, t = − 3.700, *P* = 0.021). The protein expression levels of Bax were increased and Bcl-2 were decreased in miR-383 inhibitor plus si-WNT2 group compared with that in miR-383 inhibitor plus si-NC group (Bax: ANOVA, F(7,40) = 7.013, *P* = 0.001; LSD test, t = − 2.978, *P* = 0.041. Bcl-2: ANOVA, F_(7,40)_ = 8.544, *P* = 0.000; LSD test, t = 3.077, *P* = 0.037). These results demonstrated that downregulation of miR-383 or upregulation of Wnt2 increased the expression levels of CREB, BDNF and Bcl-2, while decreased the expression levels of Bax in CUMS-induced rats.

### Downregulation of miR-383 or upregulation of Wnt2 attenuated inflammatory response in CUMS-induced rats

**Figure 7:**
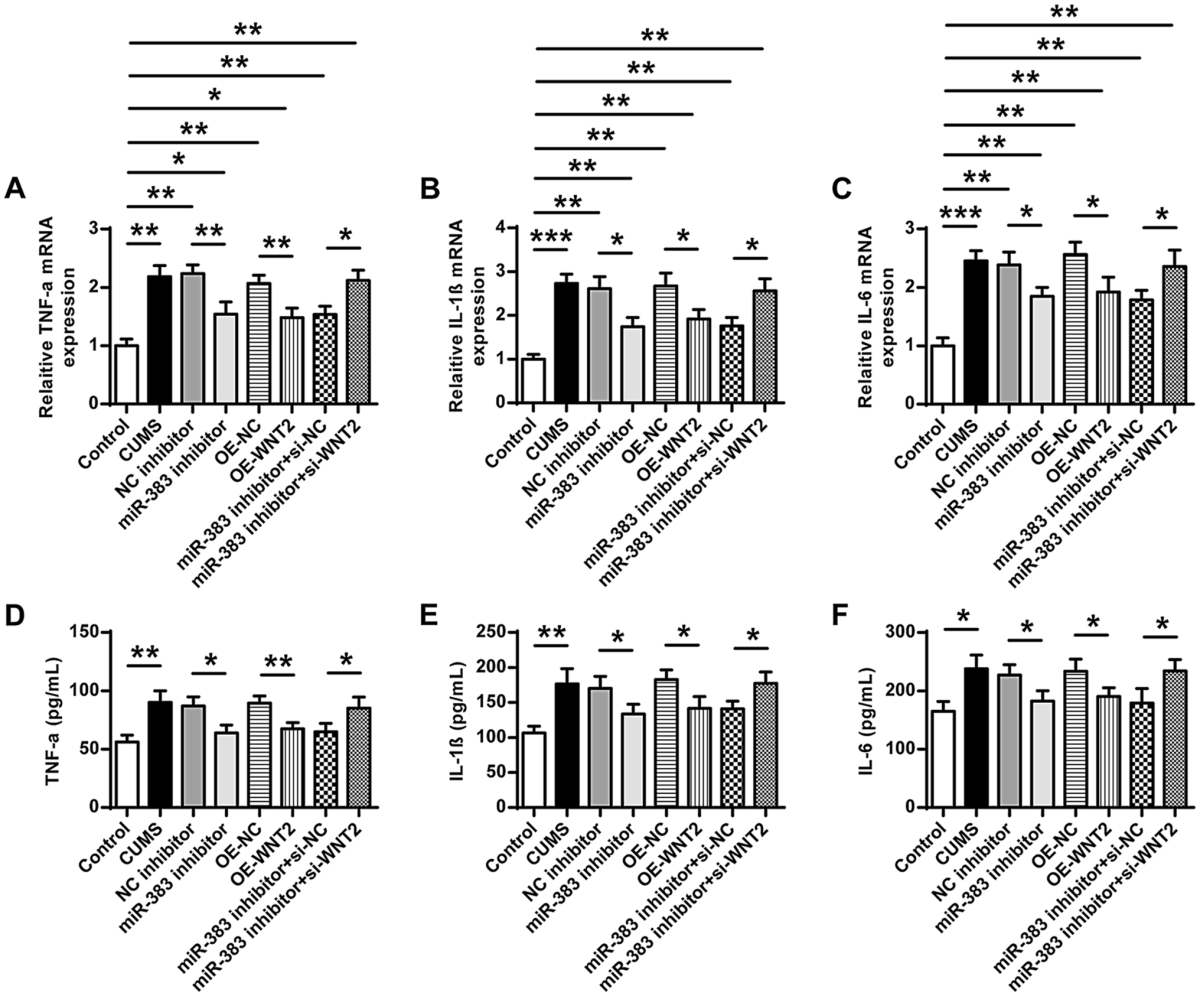
Downregulation of miR-383 or upregulation of Wnt2 attenuated inflammatory response in CUMS-induced rats. (**A**–**C**) The mRNA levels of TNF-α (**A**), IL-1β (**B**) and IL-6 (**C**) in hippocampal tissues of rats in different groups were evaluated by qRT-PCR. (**D**–**F**) The protein concentrations of TNF-α (**D**), IL-1β (**E**) and IL-6 (**F**) in hippocampal tissues of rats in different groups were evaluated by ELISA assy. N = 6. Data were presented as mean ± SD and each experiment was repeated three times. *P < 0.05, **P < 0.01, ***P < 0.001.

The expression of inflammatory cytokines including TNF-α, IL-1β and IL-6 in hippocampi tissues of different groups were evaluated by qRT-PCR (Fig. 7A–C) and ELISA assay (Fig. 7D–F). Compared with the control group, the expression levels of TNF-α were increased in the CUMS group (Fig. 7A, ANOVA, F_(7,40)_ = 22.577, *P* = 0.000; LSD test, t = − 9.292, *P* = 0.001; Fig. 7D, ANOVA, F_(7,40)_ = 10.043, *P* = 0.000; LSD test, t = − 5.067, *P* = 0.007), IL-1β (Fig. 7B, ANOVA, F_(7,40)_ = 21.971, *P* = 0.000; LSD test, t = − 12.706, *P* = 0.000; Fig. 7E, ANOVA, F_(7,40)_ = 9.303, *P* = 0.000; LSD test, t = − 5.105, *P* = 0.007) and IL-6 (Fig. 7C, ANOVA, F_(7,40)_ = 18.889, *P* = 0.000; LSD test, t = − 11.380, *P* = 0.000; Fig. 7F, ANOVA, F_(7,40)_ = 6.862, *P* = 0.001; LSD test, t = − 4.333, *P* = 0.012). Compared with the NC inhibitor group, the expression levels of TNF-α were decreased in the miR-383 inhibitor group (Fig. 7A, ANOVA, F_(7,40)_ = 22.577, *P* = 0.000; LSD test, t = 4.737, *P* = 0.009; Fig. 7D, ANOVA, F_(7,40)_ = 10.043, *P* = 0.000; LSD test, t = 3.885, *P* = 0.018), IL-1β (Fig. 7B, ANOVA, F_(7,40)_ = 21.971, *P* = 0.000; LSD test, t = 4.393, *P* = 0.012; Fig. 7E, ANOVA, F_(7,40)_ = 9.303, *P* = 0.000; LSD test, t = 2.856, *P* = 0.046) and IL-6 (Fig. 7C, ANOVA, F_(7,40)_ = 18.889, *P* = 0.000; LSD test, t = 3.486, *P* = 0.025; Fig. 7F, ANOVA, F_(7,40)_ = 6.862, *P* = 0.001; LSD test, t = 3.088, *P* = 0.037). The expression levels of TNF-α were decreased in the OE-Wnt2 group in comparison to that in OE-NC group (Fig. 7A, ANOVA, F_(7,40)_ = 22.577, *P* = 0.000; LSD test, t = 4.762, *P* = 0.009; Fig. 7D, ANOVA, F_(7,40)_ = 10.043, *P* = 0.000; LSD test, t = 4.734, *P* = 0.009), IL-1β (Fig. 7B, ANOVA, F_(7,40)_ = 21.971, *P* = 0.000; LSD test, t = 3.569, *P* = 0.023; Fig. 7E, ANOVA, F_(7,40)_ = 9.303, *P* = 0.000; LSD test, t = 3.306, *P* = 0.030) and IL-6 (Fig. 7C, ANOVA, F_(7,40)_ = 18.889, *P* = 0.000; LSD test, t = 3.361, *P* = 0.028; Fig. 7F, ANOVA, F_(7,40)_ = 6.862, *P* = 0.001; LSD test, t = 2.931, *P* = 0.043). Compared with the miR-383 inhibitor plus si-NC group, the expression levels of the three cytokines were increased in the miR-383 inhibitor plus si-Wnt2 group (TNF-α, Fig. 7A, TNF-α, ANOVA, F_(7,40)_ = 22.577, *P* = 0.000; LSD test, t = − 4.473, *P* = 0.011; Fig. 7D, ANOVA, F_(7,40)_ = 10.043, *P* = 0.000; LSD test, t = − 2.916, *P* = 0.043; IL-1β, Fig. 7B, ANOVA, F_(7,40)_ = 21.971, *P* = 0.000; LSD test, t = − 4.181, *P* = 0.014; Fig. 7E, ANOVA, F_(7,40)_ = 9.303, *P* = 0.000; LSD test, t = − 3.275, *P* = 0.031; IL-6, Fig. 7C, ANOVA, F_(7,40)_ = 18.889, *P* = 0.000; LSD test, t = − 3.014, *P* = 0.039; Fig. 7F, ANOVA, F_(7,40)_ = 6.862, *P* = 0.001; LSD test, t = − 3.039, *P* = 0.042). These results demonstrated that downregulation of miR-383 or upregulation of Wnt2 could significantly attenuate inflammatory injury in hippocampal tissues of CUMS-induced rats.

### Downregulation of miR-383 or upregulation of Wnt2 increased the expression levels of neurotransmitters in CUMS-induced rats

**Figure 8:**
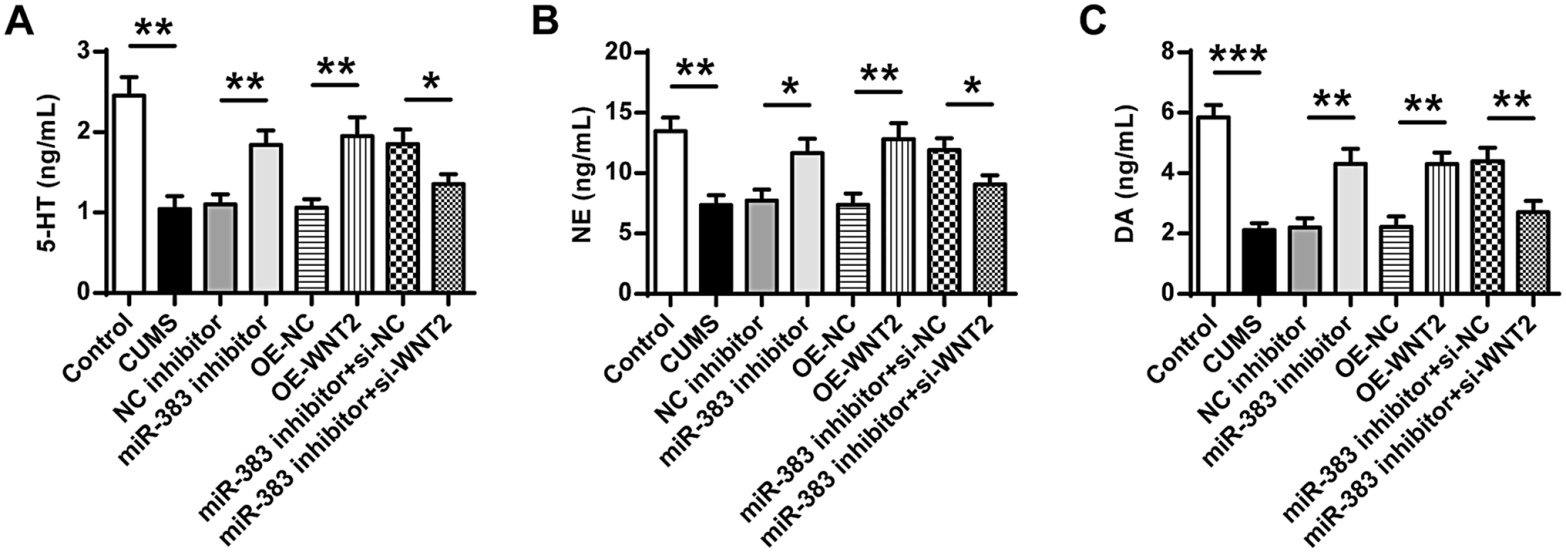
Downregulation of miR-383 or upregulation of Wnt2 increased the expression levels of neurotransmitters in CUMS-induced rats. The protein concentrations of serotonin (5-HT) (**A**), norepinephrine (NE) (**B**) and dopamine (DA) (**C**) in hippocampal tissues of rats in different groups were evaluated by ELISA assay. N = 6. Data were presented as mean ± SD and each experiment was repeated three times. *P < 0.05, **P < 0.01, ***P < 0.001.

Finally, the expression levels of neurotransmitters including serotonin (5-HT) (Fig. 8A), norepinephrine (NE) (Fig. 8B) and dopamine (DA) (Fig. 8C) in the hippocampal tissues of different groups were determined by ELISA assay. The results indicated that the expression levels of three neurotransmitters were decreased in CUMS group compared with that in the control group (5-HT, ANOVA, F_(7,40)_ = 26.643, *P* = 0.000; LSD test, t = 8.707, *P* = 0.001; NE, ANOVA, F_(7,40)_ = 18.782, *P* = 0.000; LSD test, t = 7.642, *P* = 0.002; DA, ANOVA, F_(7,40)_ = 39.531, *P* = 0.000; LSD test, t = 13.505, *P* = 0.000) (*p* < 0.01). The expression levels of 5-HT, NE and DA in miR-383 inhibitor group were increased compared with that in NC inhibitor group (5-HT, ANOVA, F_(7,40)_ = 26.643, *P* = 0.000; LSD test, t = − 5.826, *P* = 0.004; NE, ANOVA, F_(7,40)_ = 18.782, *P* = 0.000; LSD test, t = − 4.513, *P* = 0.011; DA, ANOVA, F_(7,40)_ = 39.531, *P* = 0.000; LSD test, t = − 6.347, *P* = 0.003). The expression levels of three neurotransmitters in OE-Wnt2 group were increased compared with that in OE-NC group (5-HT, ANOVA, F_(7,40)_ = 26.643, *P* = 0.000; LSD test, t = − 5.969, *P* = 0.004; NE, ANOVA, F_(7,40)_ = 18.782, *P* = 0.000; LSD test, t = − 5.815, *P* = 0.004; DA, ANOVA, F_(7,40)_ = 39.531, *P* = 0.000; LSD test, t = − 7.001, *P* = 0.002). Compared with miR-383 inhibitor plus si-NC group, the expression levels of three neurotransmitters were decreased in the miR-383 inhibitor plus si-Wnt2 group (5-HT, ANOVA, F_(7,40)_ = 26.643, *P* = 0.000; LSD test, t = 3.936, *P* = 0.017; NE, ANOVA, F_(7,40)_ = 18.782, *P* = 0.000; LSD test, t = 3.879, *P* = 0.018; DA, ANOVA, F_(7,40)_ = 39.531, *P* = 0.000; LSD test, t = 4.986, *P* = 0.008). These results demonstrated that downregulation of miR-383 or upregulation of Wnt2 could efficiently enhance the expression levels of neurotransmitter of hippocampal tissues in CUMS-induced rats.

## Discussion

It was predicted that MDD will become the major killer of human health except for cardiovascular diseases by 2030^20^. Therefore, it is necessary to develop new and efficient therapies to prevent the progression of MDD. In the last decades, studies have identified miRNAs that could be considered as potential therapeutic targets for the treatment of MDD. The expression of miR-124 in serum was significantly upregulated along with depression and antidepressants, and might be a potential indicator for depression status^21^. It was reported that miR-146a/b-5, miR-425-3p and miR-24-3p were dysregulated with antidepressant response through regulating MAPK/Wnt-system genes and might act as efficient biomarkers of treatment response^22^. MiR-221 has been identified to be closely associated with the progression of depression via targeting the Wnt2/CREB/BDNF axis in hippocampal neurons in a CUMS model^19^. The potential contribution of miR-124-3p in the pathophysiology of MDD has been reported and it indicated that miR-124-3p might act as a novel target for drug development and a biomarker for MDD pathogenesis^23^. MiR-155 was significantly upregulated in serum of individuals with depression compared with those in healthy controls, and affected the development of MDD through targeting SIRT1^24^. MiR-124 ameliorated depressive-like behavior via targeting STAT3 to regulate microglial activation in MDD^25^. Despite the abnormal expression of many miRNAs in MDD, the underlying mechanisms are still not well known. Although miR-383 has been reported to participate in multiple biological processes in human cancers, little is known in MDD. In this study, we found that miR-383 was markedly upregulated in the hippocampi tissues of CUMS-induced rats, suggesting a potential acceleration effect of miR-383 in MDD.

To further explore the mechanism of miR-383 in MDD, rats were injected with miR-383 inhibitor or NC inhibitor. As expected, miR-383 inhibitor could efficiently inhibit the apoptosis of hippocampal neurons and inflammatory response, and increase the expression levels of GFAP, BDNF, CREB as well as neurotransmitters, then attenuated neural injury in CUMS-induced rats. Meanwhile, Wnt2 was identified as a direct target of miR-383, and luciferase reporter assay confirmed their interaction. Wnt2 often acts as the target gene of miRNAs to regulate the progression of human diseases. MiRNA-199a/b-5p promoted imatinib efficacy through inhibiting the Wnt2 signaling pathway-mediated protective autophagy in imatinib-resistant chronic myeloid leukemia cells^26^. MiR-199a-5p regulated cell proliferation and morphology of smooth muscle cells via targeting the Wnt2 pathway^27^. Downregulation of miR-30a-3p/5p promoted cell proliferation of esophageal squamous cell carcinoma through targeting Wnt2 and Fzd2^28^. Overexpression of miR-214 promoted the development of human osteosarcoma via regulating the Wnt/β-catenin signaling pathway^29^. In MDD, the expression levels of Wnt2 are significantly increased by different classes of antidepressant treatments^30^. However, the regulation of Wnt2 in MDD is unknown. Only one study demonstrated that miR-221 promoted the progression of MDD through targeting the Wnt2/CREB/BDNF axis^19^. Here, we demonstrated that miR-383 affected the depressive-like behavior in CUMS-induced rats through directly targeting Wnt2, and silencing of Wnt2 could significantly reverse miR-383 inhibitor caused protective effects on the apoptosis of hippocampal neurons, inflammatory response, the expression levels and neurotransmitters in hippocampal tissues of CUMS-induced rats. The results demonstrated that the function of miR-383 in MDD was closely mediated by Wnt2.

In addition, increasing evidence has indicated that the progression of MDD is associated with the increased activity of the immune system^31,32^. Moreover, a large number of clinical trials have demonstrated anti-inflammatory treatment exerts better effects in depression^33^. For example, it was found that the antidepressant activity of celecoxib might be closely related to its capability of reducing IL-6 concentrations, and celecoxib might be a safe and effective antidepressant^34^. The combination of fluoxetine and celecoxib has been found to exert a significant superiority over fluoxetine alone in major depression treatment^35^. These findings suggest that well known of anti-inflammatory mechanisms in MDD may contribute to identify and develop specific antidepressants. In the present study, we found that downregulation of miR-383 or overexpression of Wnt2 could significantly decreased the expression levels of inflammatory cytokines including TNF-α, IL-1β and IL-6, while co-transfection of miR-383 inhibitor and si-Wnt-2 obviously reversed the inhibitory effect of miR-383 inhibitor on the expression of inflammatory cytokines in hippocampal tissues of CUMS-induced rats. Therefore, miR-383 might act as an anti-depression target for identifying and developing novel antidepressants.

Moreover, previous studies have suggested that the brain neurotransmitters including serotonin (5-HT), norepinephrine (NE) and dopamine (DA) are incriminated in the progression of MDD, and dysfunction of these neurotransmitters may lead to the genesis of MDD^36^. Antidepressants targeting these monoamines can directly affect the functional tone of these circuits, notably in limbic and front cortical areas, confirming that this action plays essential roles in the treatment of MDD^37^. These findings suggest that targeting these brain neurotransmitters also may be potential drug targets for MDD treatment. Here, our study revealed that the expression of 5-HT, NE and DA were all dramatically downregulated in the hippocampal tissues of CUMS-induced rats, and downregulation of miR-383 or overexpression of Wnt2 significantly increased their expression levels, while silencing of Wnt2 obviously decreased the expression levels of these neurotransmitters induced by inhibitor of miR-383 or overexpression of Wnt2.

In addition, the network of miRNA-mRNA regulatory machinery is complex and difficult to be determined by exploring individual pairs of interactions^38^. A single miRNA can target hundreds of miRNAs/mRNAs and influence the expression of many genes involved in a functional interacting pathway^39^. Therefore, there might be several potential targets of miR-383 that are associated with the progression of depression, which needs to be explored in the future.

## Conclusion

In summary, our results demonstrated that downregulation of miR-383 could efficiently reduce depression-like behavior in CUMS-induced rats through directly promoting the expression of Wnt2, providing a novel therapeutic target for MDD treatment.

## Materials and methods

### Animals

A total of 60 adult male SD rats (approximately 180–220 g, 6-week-old) were obtained from Laboratory Animal Department (Qingdao University, China). Rats were kept at 24 ± 2 °C with 12 h day/night cycle and free access to food and water. All animal studies were approved by the Animal Care Committee of Qingdao Mental Health Center, Qingdao University and operated according to the American Animal Protection Legislation. The study was carried out in compliance with the ARRIVE guidelines.

### The construction of depressive disorder rat model

The depressive disorder rat model was induced by chronic unpredictable mild stress (CUMS) as previously described^40^. Briefly, rats were randomly divided into two groups (n = 6): the control group and MDD group (CUMS). For CUMS group, rats were stimulated with random stress including ice-water swimming for 5 min, food deprivation for 24 h, water deprivation for 24 h, stimulating tail for 1 min, turning night into day, shaking for 15 min (once/s), and wrap restraint (5 min/time) every day for 21 d. And for the control group, rats were fed without any induction.

### Stereotactic injection of rat hippocampus

Stereotactic injection of rat hippocampus was performed as previously described^41^. In brief, rats were anesthetized by 30 mg/kg sodium pentobarbital via intraperitoneal injection. After disinfected by iodophor, the scalp of rat was cut open along the midline of calvarium, and the anterior fontanel was ignited by 3% hydrogen peroxide. According to the rat brain atlas^42^, the position was: 3.3 mm backward, 1.8 mm to the right and the left of the anterior fontanel, and 2.6 mm downward the surface of skull, then a hole was drilled via a mini electric drill. A microsyringe (10 μL) was fixed on the stereotaxic instrument, by which the rats of each group (n = 6) were injected with 2 μL normal saline, miR-383 inhibitor, NC inhibitor, OE-Wnt2 (overexpression of Wnt2), OE-NC, miR-383 inhibitor + si-NC, miR-383 inhibitor + si-Wnt2 with 0.25 μL/min. MiR-383 inhibitor, NC inhibitor, OE-Wnt2, si-WNT2 and corresponding negative controls (OE-NC and si-NC) were all purchased from Shanghai Genechem Co., Ltd. (Shanghai, China). Rats of the control group received no treatment. Then the skin was disinfected by iodine and sutured. After injection, the needle stayed in the same spot for 5 min before withdrawing. Subsequently, rats were kept warm until full recovery from anesthesia. Rats were allowed to recover for at least 1 week before behavioral experiments or expression evaluation. Rats were recovered for 1 week before exposure to CUMS.

### Ethological examination

The sugar preference test was performed as previously described^43^. In brief, rats in each group underwent water and food deprivation for 24 h before the experiment. A bottle of 1% sucrose solution (200 mL) and a bottle of purified water (200 mL) were provided for each rat, the two bottles were the same and without leakage. The consumption of sucrose solution and purified water of each rat in 1 h was measured. Sugar preference rate (%) = the consumption of sucrose solution (mL)/the consumption of sucrose solution and purified water (mL) × 100%.

The refuge island tests were performed as previously described^44^. Briefly, the refuge island was randomly set in one of the four quadrants of water maze, the water was 10 mm over the island, and the temperature was at 22–25 °C. Swimming training was conducted with rats during a fixed time each day, every rat was trained in the different quadrant. Rats haven’t found the refuge island in 120 s were placed on the platform for 10 s, then the next training was carried on, the training was continued for 4 d. The test was completed until all the rats could find the refuge island in approximately the same time. The test was conducted before and after the models were established for 3 d (6 times/d), escape latency (EL) was the time that the rats found the platform in 120 s, and the test score in the last 3 d was recorded as the spatial memory score.

Open field tests, including walking distance, central activity time, erect frequency, social grooming and EL, were performed as previously described^45^. This test was conducted during a same time period each day. The rats were placed on the center grid of the field by lifting tails and their ethology was analyzed by Dr. Rat video analysis system (Shanghai Mobile Datum information technology Co., Ltd., Shanghai, China). The field (length of 120 cm, width of 120 cm and height of 35 cm) with a black bottom was set in a quiet room. The open field was washed by 75% ethanol after the test with the last rat was over, and the test of next rat was started after ethanol was volatilized. All experiments in each group were performed simultaneously. After the behavioral tests, the hippocampus tissues of rats from each group were removed and used for the subsequent biochemistry and morphological evaluation.

### Nissl’s staining

The relative morphological changes of hippocampal neurons were evaluated using Nissl’s staining as previously described^46^. In brief, the paraffin sections of hippocampal tissues with 4 μm in thickness were soaked in xylene twice, each time for 10 min, and successively immersed in absolute ethanol, 95% ethanol and 70% ethanol, each concentration for 2 min, then washed with distilled water. Next, the sections were added with 1% toluidine blue for 6 min, separated by 70% ethanol (2 min) and 95 ethanol (3–5 min). When the nuclei turned bright blue and the background was colorless under a microscope, the sections were soaked in 100% ethanol for three times (1 min/time), permeabilized by coagulating agent twice, 5 min/time, and sealed by neutral balsam. The loss of hippocampal neurons was evaluated by a microscope in six non-overlapping fields (200 × magnification).

### TUNEL staining

Apoptosis rate of hippocampal neurons in different groups was evaluated by TUNEL staining with ApopTag Kit-S7100 (Roche, Basel, Switzerland) as previously described^47^. The dewaxed sections of hippocampal tissues with 4 μm in thickness were added with proteinase solution (20 mg/L) for 15 min and 50 μL TUNEL reagent, and incubated in a wet box at 37 °C without light exposure for 60 min. Subsequently, the sections were appended with anti-fluorescein containing horseradish peroxidase (HRP) and incubated in a wet box at 37 °C without light exposure for 30 min, and the sections were developed by diaminobenzidine (DAB) for 5–10 min, counterstained by hematoxylin, dehydrated and permeabilized, then sealed by neutral balsam. The sections were captured by IP lab7.0 and IX70 inverted microscope (Olympus, Tokyo, Japan) at 200 × magnification. The positive cells were counted by blind method, mean value of the number of positive cells in per unit area was recorded.

### Immunohistochemical staining

Immunohistochemical staining assay was carried out according to the previous study^48^. In brief, the samples of hippocampal tissues were embedded by paraffin, sectioned into 4 μm in thickness and toasted at 70 °C for 15 min, dehydrated by gradient ethanol, inactivated by H_2_O_2_, and incubated by normal goat serum for 15 min. Then the primary antibody against GFAP (1:1,000, Abcam Inc., Cambridge, MA, USA) was added and incubated overnight at 4 °C. The sections were incubated with biotin-labelled secondary antibody for 30 min. After rinsed by PBS, the sections were incubated by HRP-labelled streptavidin solution for 20 min, developed by DAB (ZSGB-Bio, Beijing, China) for 3–5 min and counterstained by hematoxylin for 5 min, then dehydrated, permeabilized by xylene, and sealed. PBS was taken as the blank group to replace the primary antibody. The GFAP-positive control images that were provided by the kits of Fuzhou Maxim Biotechnology Co., Ltd. were considered as the positive control. Finally, pictures were captured using a Leica fluorescence microscope (magnification, 200x). Five fields of view were randomly selected, and the positive expression of GFAP was observed.

### ELISA assay

Hippocampal tissues were diluted with saline solution and centrifuged at 3,000 rpm/min at 4 °C for 25 min. The expression levels of tumor necrosis factor-α (TNF-α), interleukin-1β (IL-1β), IL-6, 5-hydroxytryptamine (5-HT), noradrenaline (NE) and dopamine (DA) in the supernatant were detected using corresponding ELISA kits (Elisa Biotech Co., Ltd., Shanghai, China) following the manufacturer’s instructions. The absorbance at 450 nm was measured in 10 min. The standard curve was graphed using Curve Expert 1.3 analysis software and the content of samples was calculated as previously described^49^.

### qRT-PCR

Total RNAs were extracted from tissues or cultured cells by Trizol agent, and approximately 1.2 μg RNA was reversely transcribed into cDNA using the reverse transcription kit (K1621, Fermentas, Maryland, NY, USA). Then quantitative real time PCR was performed by fluorescence quantitative PCR kits (Takara Biotechnology Ltd., Dalian, China) and ABI 7500 RT-qPCR instrument. The relative expression changes of targets were analyzed by 2^-∆ ∆Ct^ method^50^. The relative expression levels of miRNAs and mRNAs were normalized to U6 snRNA and GAPDH, respectively. The primers used in this study were listed in Table 1.

**Table 1:** Sequences of primers used in this study.

Gene	Forward primer (5′–3′)	Reversed primer (5′–3′)
miR-370	TGTAACCAGAGAGCGGGATGT	TTTTGGCATAACTAAGGCCGAA
miR-383	CACGAAAGATCAGAAGGTGA	CTCAACTGGTGTCGTGGA
miR-590-3p	GCAGTGGAATGTAAGGAAGTGTGT	GCGAGCACAGAATTAATACGACTC
miR-329	GGGAACACACCTGGTTAAC	CAGTGCGTGTCGTGGAGT
miR-144	GCCCCTACAGTATAGATGATGTA	GTGCAGGGTCCGAGGT
miR-136	GCGCTGGAGTGTGACAATGGTG	GTGCAGGGTCCGAGGT
miR-362-3p	CAGGGACTGAGGGCAATCGT	TTCATCGCGGTCGAGGGCGG
miR-211	TTGTGGGCTTCCCTTTGTCATCCT	TGCTGTGGGAAGTGACAACTGA
WNT2	GTTCTTGAAACAAGAGTGCAAGTG	CCCATTGTACTTCCTCCAGAGATA
GFAP	CTCAATGCTGGGTTCAAGGAGA	GACGCAGCGTCTGTGAGGTC
CREB	GTGCCAGCCTTTCCTTACAC	CACAAACCCACTGATGAACG
BDNF	TGCTGGATGAGGACCAGAAG	TTCCTCCAGCAGAAAGAGCA
TNF-α	TCAGCCGATTTGCCATTTCAT	ACACGCCAGTCGCTTCACAGA
IL-1β	GTCCTTTCACTTGCCCTCAT	CAAACTGGTCACAGCTTTCGA
IL-6	AAATGCCTCGTGCTGTCTGACC	GGTGGGTGTGCCGTCTTTCATC
GAPDH	GAAGAGTGGGTGTCGCTGTT	CTGCCGTCTGGAAAAACCT
U6	TGCGGGTGCTCGCTTCGCAGC	CCAGTGCAGGGTCCGAGGT

### Western blot

Total proteins of hippocampal tissues were extracted using RIRP lysis buffer and the protein concentration was determined by BCA kit. Western blot assay was performed as previously described with minor modification^51^. In brief, approximately 25 μg protein was separated by 12% SDS-PAGE and transferred onto PVDF membranes. After blocking with 5% skim milk, the membranes were incubated with primary antibodies at ratio 1:1,000 including Wnt2, Bcl-2, Bax, GFAP, BDNF, p-CREB, CREB overnight at 4 °C, with GAPDH (1:5,000) as the internal reference. All primary antibodies were purchased from Abcam Inc., Cambridge, MA, USA. Then HRP-conjectured secondary antibody was then added to incubate the membranes for 1 h. Finally, the membranes were exposed by ECL kit, and gray values were analyzed using Image J.

### Cell culture

Human embryonic kidney cell line HEK 293 T and PC12 cells were purchased from Life Technologies Corporation, ThermoFisher Scientific (Grand Island, NY). Cells were cultured in DMEM medium containing 10% fetal bovine serum, 100 mg/L penicillin and 100 mg/L streptomycin at 37 °C in humidified air containing 5% CO_2_.

### Luciferase reporter assay

Luciferase reporter assay was performed as previously described^52^. Briefly, the 3′-UTR fragments of WNT2 containing the miR-383 binding site (WT) or the corresponding mutant (MUT) were inserted into pmirGLO Dual-Luciferase miRNA Target Expression Vector (Promega, Madison, WI, USA) at the downstream of the firefly luciferase gene. The recombinant luciferase reporter plasmids were co-transfected with miR-383 mimics or NC mimics into 293 T/PC12 cells using Lipofectamine 3000 kit following the manufacturer’s instructions. MiR-383 mimics and corresponding negative control (NC mimics) were purchased from Shanghai Genechem Co., Ltd. (Shanghai, China). After transfection for 48 h, cells were collected and the relative luciferase activity was detected by Dual-Luciferase Reporter Assay System.

### Statistical analyses

All data were presented as the means ± standard deviation (SD). Statistical analysis was performed using the SPSS 21.0 software. Comparisons between two groups were analyzed using t test. Comparisons among multiple groups were performed using one-way analysis of ANOVA followed by a post hoc LSD test if data conformed to normality and homogeneity of variance (tested by the Kolmogorov–Smirnov test). The correlation between WNT2 and miR-383 was tested with Pearson’s correlation. *P* < 0.05 was considered as the significant threshold.

### Ethics approval

All animal studies were approved by the Animal Care Committee of Qingdao Mental Health Center, Qingdao University and they were operated according to the American Animal Protection Legislation. The study was carried out in compliance with the ARRIVE guidelines.

## Supplementary Information

Supplementary Figures.

## Data Availability

The datasets used and/or analyzed during the current study are available from the corresponding author on reasonable request.
