# Supplementary material for: Downregulation of miR-383 reduces depression-like behavior through targeting Wnt family member 2 (Wnt2) in rats
Source: Sci Rep. 2021 Apr 29;11:9223. doi: 10.1038/s41598-021-88560-6 (PMC8085118; doi:10.1038/s41598-021-88560-6)

Downregulation of miR-383 reduces depression-like behavior through targeting Wnt family member 2 (Wnt2) in rats

Running title: miR-383/Wnt2 in CUMS-induced rats

Shanshan Liu[2], Qing Liu[2], Yanjie Ju[3], Lei Liu[*1]

[1]Department of Psychiatric, Qingdao Mental Health Center, Qingdao University, Qingdao City, Shandong Province, 266000, PR. China.

[2]Department of Clinical Psychology, Qingdao Mental Health Center, Qingdao University, Qingdao City, Shandong Province, 266000, PR. China.

[3]Department of Open Room, Qingdao Mental Health Center, Qingdao University, Qingdao City, Shandong Province, 266000, PR. China.

Correspondence author: * Lei Liu, Department of Psychiatric, Qingdao Mental Health Center, Qingdao University, No.299 Nanjing Road, Qingdao City, Shandong Province, 266000, PR. China.E-mail address: dt6031@163.com Tel: +86-0532-85621584

Supplementary Fig. 1 Downregulation of miR-383 reduced depression-like behaviors in normal rats. Normal rats were injected with miR-383 inhibitor or NC inhibitor. The sucrose preference (A), walking distance (B), central activity time (C), Erect frequency (D), Social grooming (E) and Escape latency (EL) (F) of rats in different groups. N = 6. Data were presented as mean ± SD and each experiment was repeated three times. * P < 0.05, ** P < 0.01.

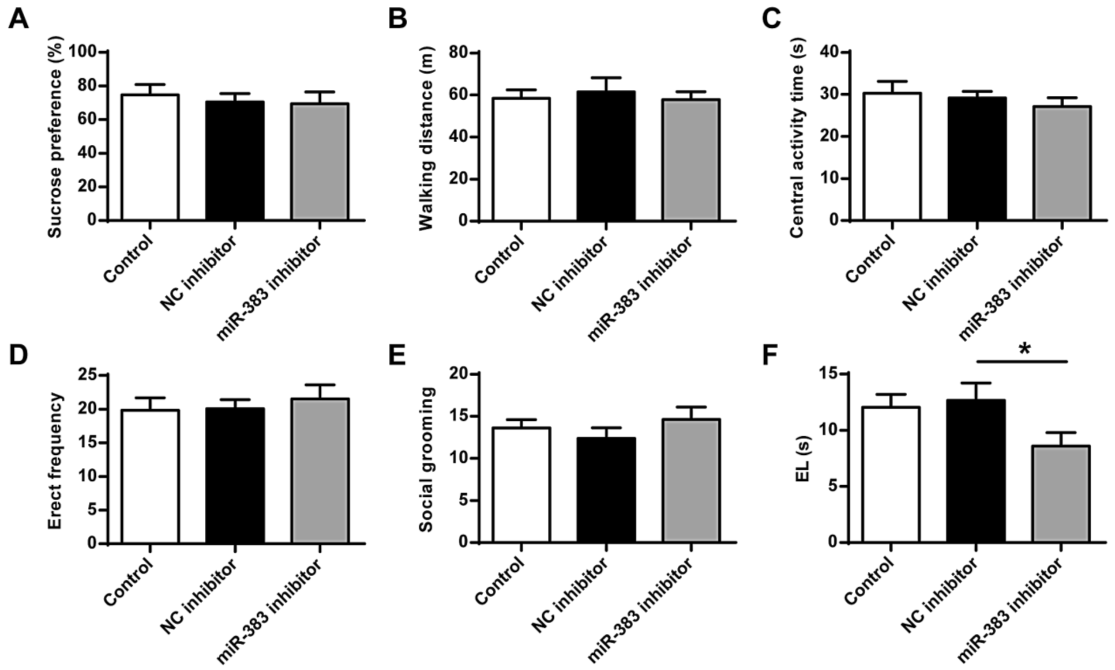

Figure 1B WNT2、GAPDH

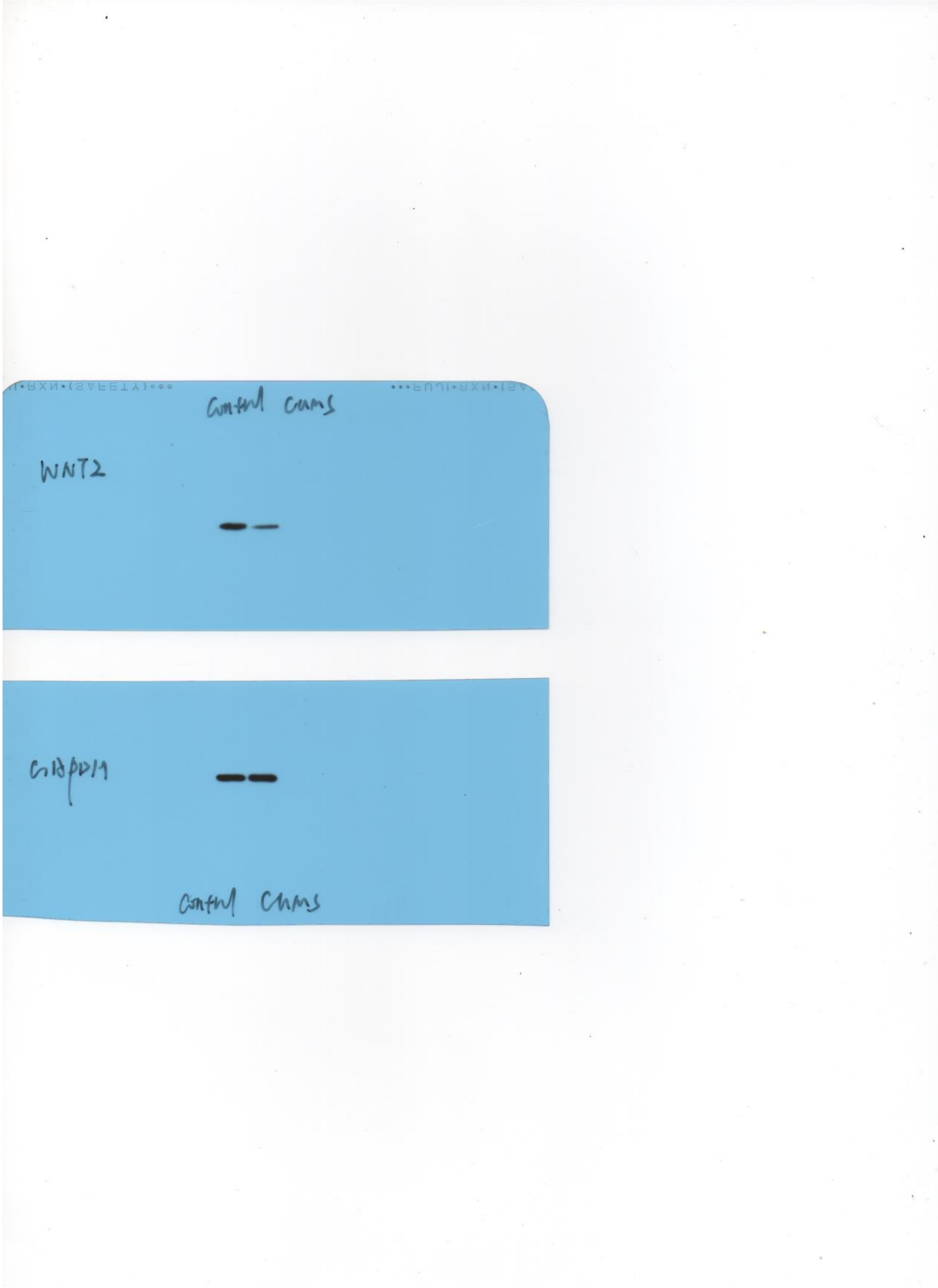

Figure 2F WNT2、GAPDH

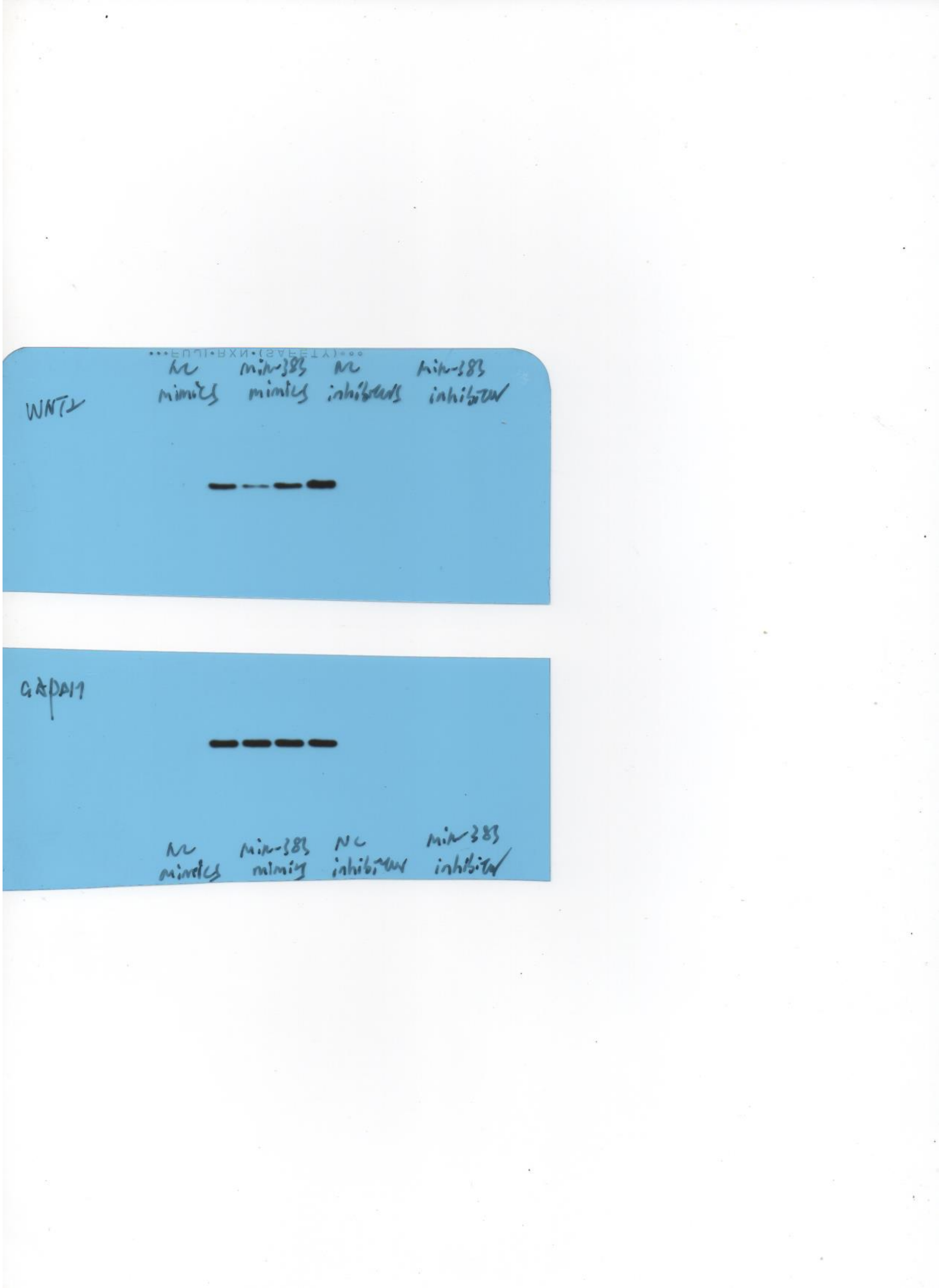

Figure 2I WNT2、GAPDH

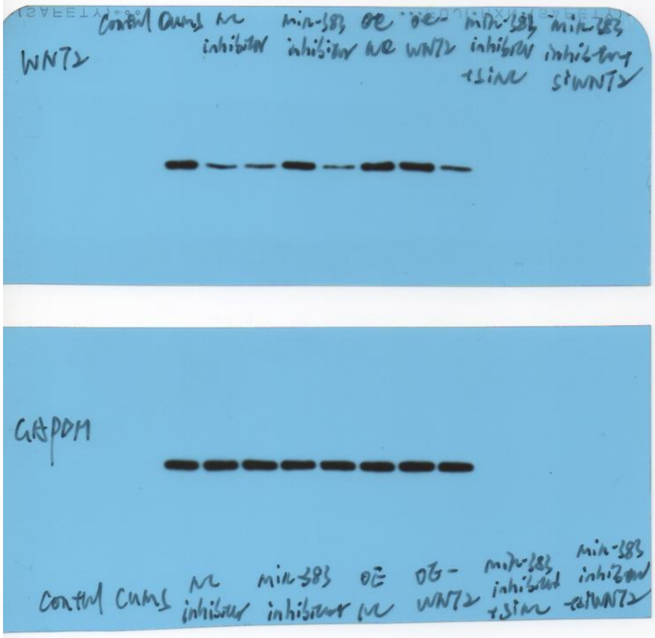

Figure 5C GFAP、GAPDH

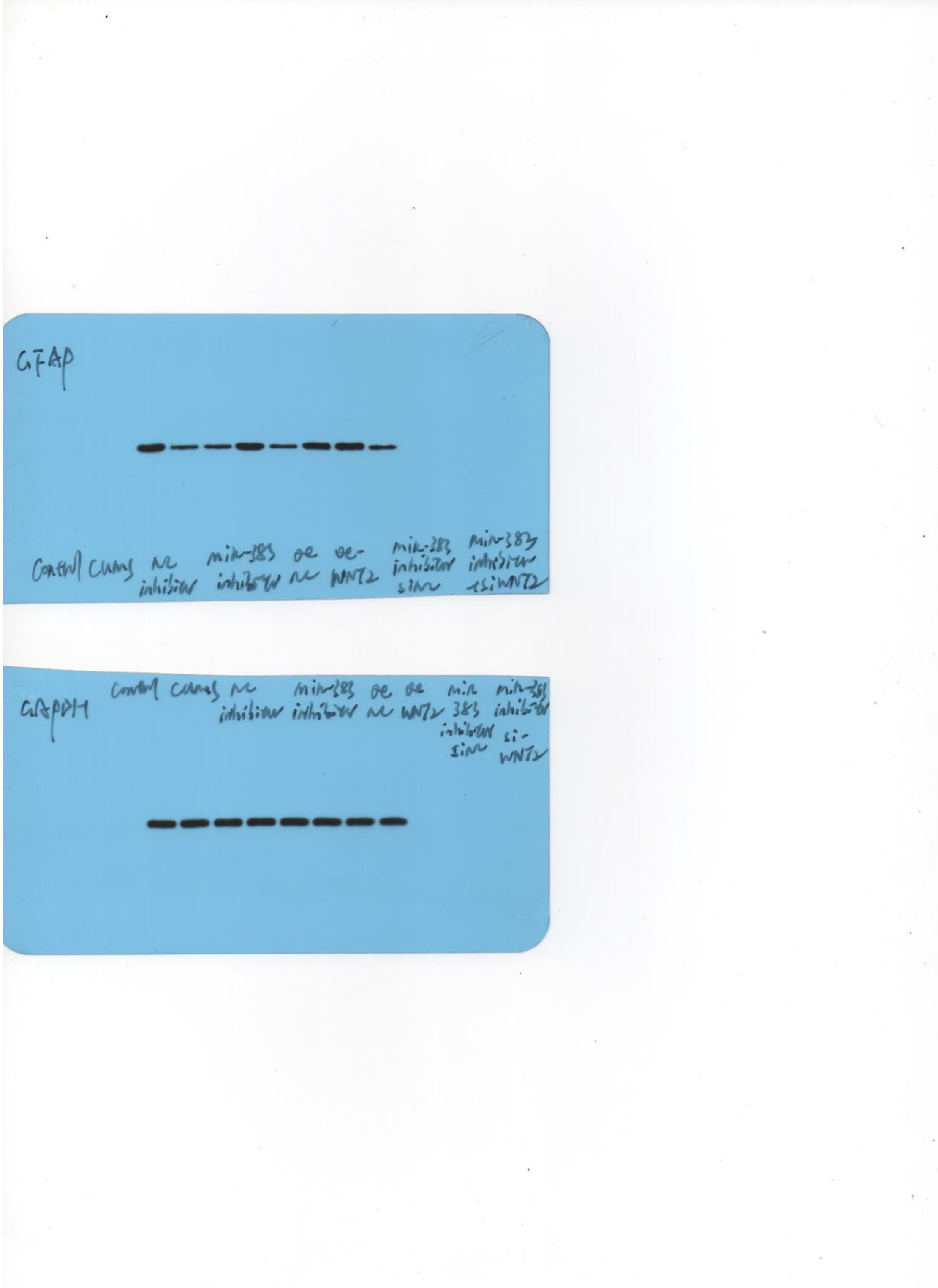

Figure 6C p-CREB、CREB、GAPDH

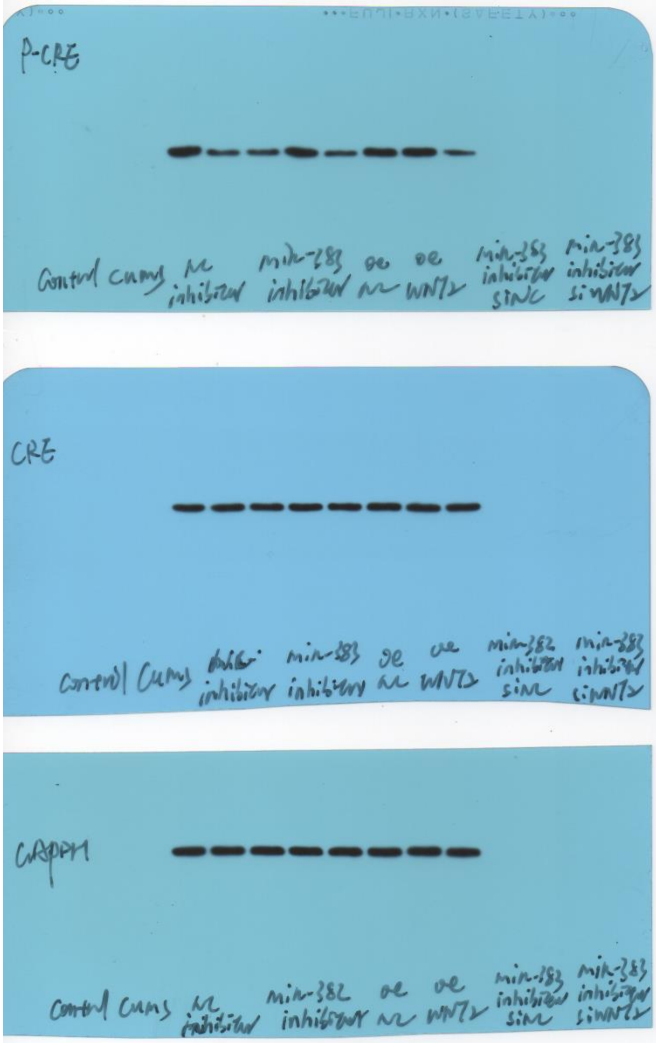

Figure 6D BDNF、GAPDH

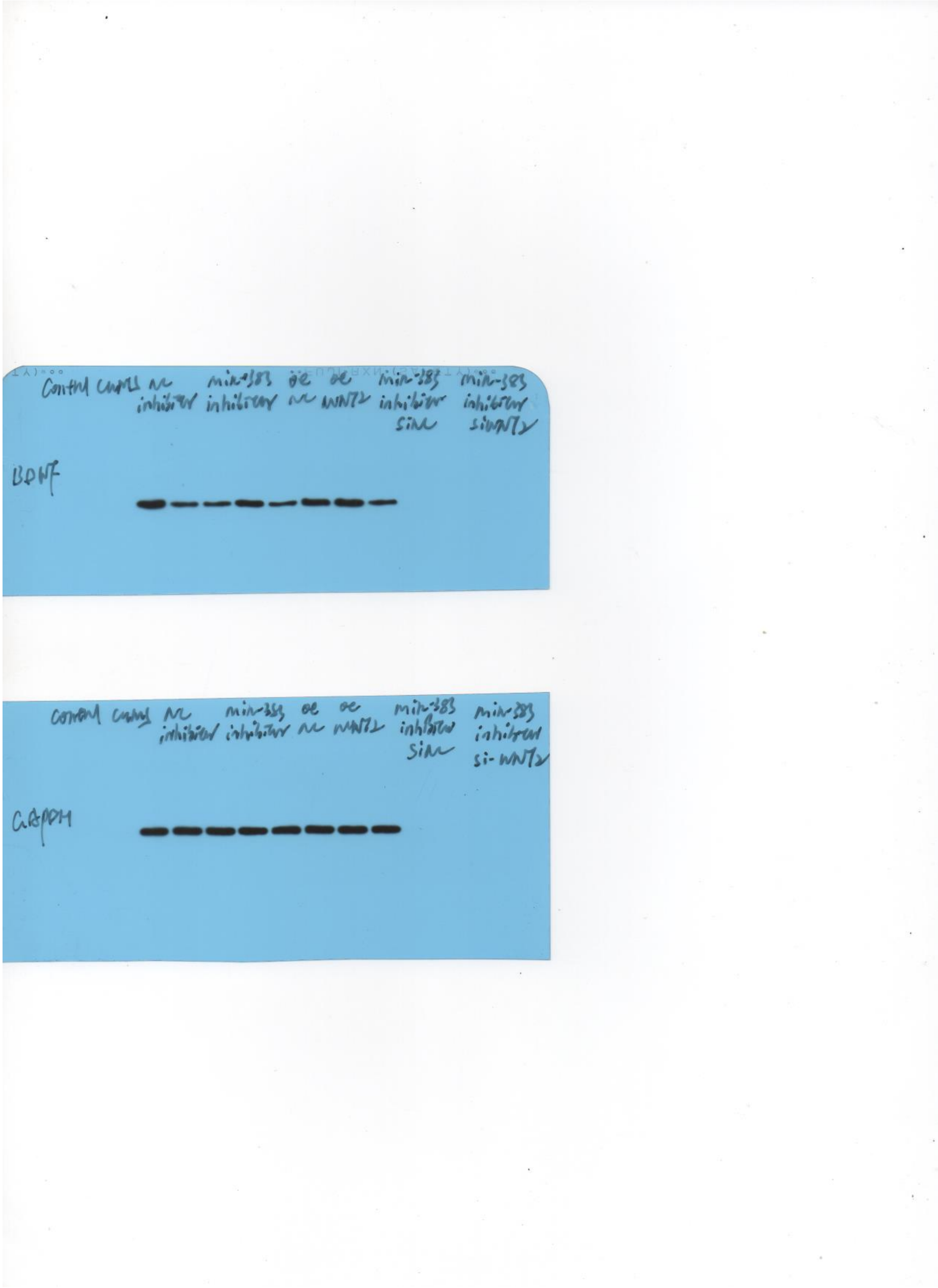

Figure 6E Bax、Bcl-2、GAPDH

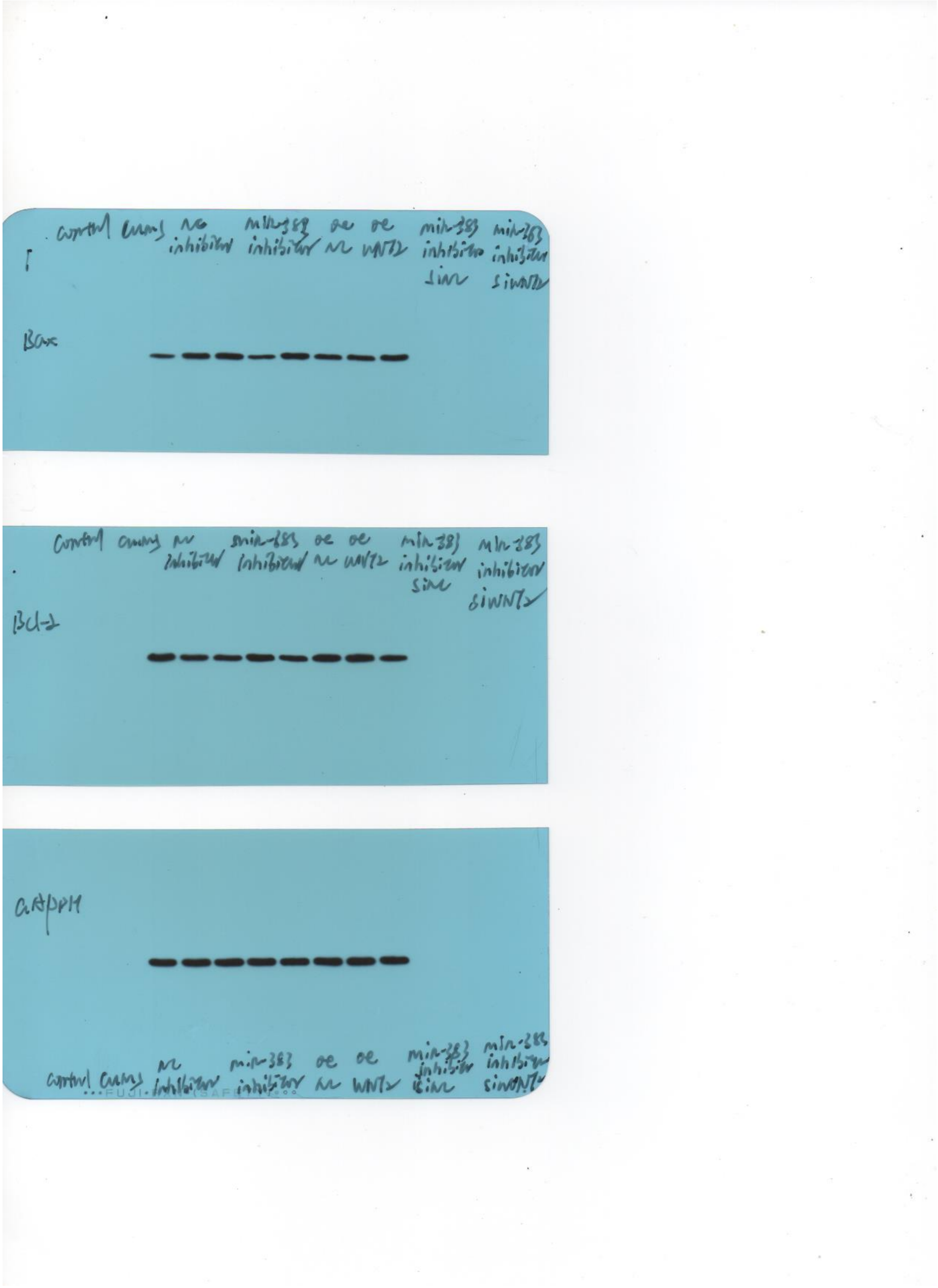

Supplement: Supplementary file 1 — Supplementary Figures. [file 41598_2021_88560_MOESM1_ESM.pdf]
